# Efficient and heritable A-to-K base editing in rice and tomato

**DOI:** 10.1093/hr/uhad250

**Published:** 2023-12-11

**Authors:** Xinbo Li, Jiyong Xie, Chao Dong, Zai Zheng, Rundong Shen, Xuesong Cao, Xiaoyan Chen, Mugui Wang, Jian-Kang Zhu, Yifu Tian

**Affiliations:** Ministry of Agriculture and Rural Affairs Key Laboratory of Gene Editing Technologies (Hainan), Institute of Crop Sciences and National Nanfan Research Institute, Chinese Academy of Agricultural Sciences, Sanya, Hainan 572024, China; Hainan Yazhou Bay Seed Lab, Sanya, Hainan 572024, China; Shanghai Center for Plant Stress Biology, CAS Center for Excellence in Molecular Plant Sciences, Chinese Academy of Sciences, Shanghai 201602, China; University of Chinese Academy of Sciences, Beijing 100049, China; Ministry of Agriculture and Rural Affairs Key Laboratory of Gene Editing Technologies (Hainan), Institute of Crop Sciences and National Nanfan Research Institute, Chinese Academy of Agricultural Sciences, Sanya, Hainan 572024, China; Hainan Yazhou Bay Seed Lab, Sanya, Hainan 572024, China; Ministry of Agriculture and Rural Affairs Key Laboratory of Gene Editing Technologies (Hainan), Institute of Crop Sciences and National Nanfan Research Institute, Chinese Academy of Agricultural Sciences, Sanya, Hainan 572024, China; Hainan Yazhou Bay Seed Lab, Sanya, Hainan 572024, China; Ministry of Agriculture and Rural Affairs Key Laboratory of Gene Editing Technologies (Hainan), Institute of Crop Sciences and National Nanfan Research Institute, Chinese Academy of Agricultural Sciences, Sanya, Hainan 572024, China; Hainan Yazhou Bay Seed Lab, Sanya, Hainan 572024, China; Institute of Advanced Biotechnology, and School of Life Sciences, Southern University of Science and Technology, Shenzhen 518055, China; Ministry of Agriculture and Rural Affairs Key Laboratory of Gene Editing Technologies (Hainan), Institute of Crop Sciences and National Nanfan Research Institute, Chinese Academy of Agricultural Sciences, Sanya, Hainan 572024, China; Ministry of Agriculture and Rural Affairs Key Laboratory of Gene Editing Technologies (Hainan), Institute of Crop Sciences and National Nanfan Research Institute, Chinese Academy of Agricultural Sciences, Sanya, Hainan 572024, China; Ministry of Agriculture and Rural Affairs Key Laboratory of Gene Editing Technologies (Hainan), Institute of Crop Sciences and National Nanfan Research Institute, Chinese Academy of Agricultural Sciences, Sanya, Hainan 572024, China; Institute of Advanced Biotechnology, and School of Life Sciences, Southern University of Science and Technology, Shenzhen 518055, China; Ministry of Agriculture and Rural Affairs Key Laboratory of Gene Editing Technologies (Hainan), Institute of Crop Sciences and National Nanfan Research Institute, Chinese Academy of Agricultural Sciences, Sanya, Hainan 572024, China; Hainan Yazhou Bay Seed Lab, Sanya, Hainan 572024, China

## Abstract

Cytosine and adenosine base editors (CBE and ABE) have been widely used in plants, greatly accelerating gene function research and crop breeding. Current base editors can achieve efficient A-to-G and C-to-T/G/A editing. However, efficient and heritable A-to-Y (A-to-T/C) editing remains to be developed in plants. In this study, a series of A-to-K base editor (AKBE) systems were constructed for monocot and dicot plants. Furthermore, nSpCas9 was replaced with the PAM-less Cas9 variant (nSpRY) to expand the target range of the AKBEs. Analysis of 228 *T*_0_ rice plants and 121 *T*_0_ tomato plants edited using AKBEs at 18 endogenous loci revealed that, in addition to highly efficient A-to-G substitution (41.0% on average), the plant AKBEs can achieve A-to-T conversion with efficiencies of up to 25.9 and 10.5% in rice and tomato, respectively. Moreover, the rice-optimized AKBE generates A-to-C conversion in rice, with an average efficiency of 1.8%, revealing the significant value of plant-optimized AKBE in creating genetic diversity. Although most of the A-to-T and A-to-C edits were chimeric, desired editing types could be transmitted to the *T*_1_ offspring, similar to the edits generated by the traditional ABE8e. Besides, using AKBEs to target tyrosine (Y, TAT) or cysteine (C, TGT) achieved the introduction of an early stop codon (TAG/TAA/TGA) of target genes, demonstrating its potential use in gene disruption.

## Introduction

Single-nucleotide polymorphism (SNP) is a common type of genetic diversity in plants and is associated with numerous agronomic traits. By creating specific SNPs, genetic improvement can be achieved, thus accelerating the breeding process [1]. Base editors are efficient tools for base substitution, e.g. ABE for A-to-G substitution [2], CBE for C-to-T substitution [3], and CGBE for C-to-A/G substitution [4–6]. At present, base conversion types produced by base editors are still limited, and A-to-T/C transversion base editors remain to be developed in plants.

**Figure 1:**
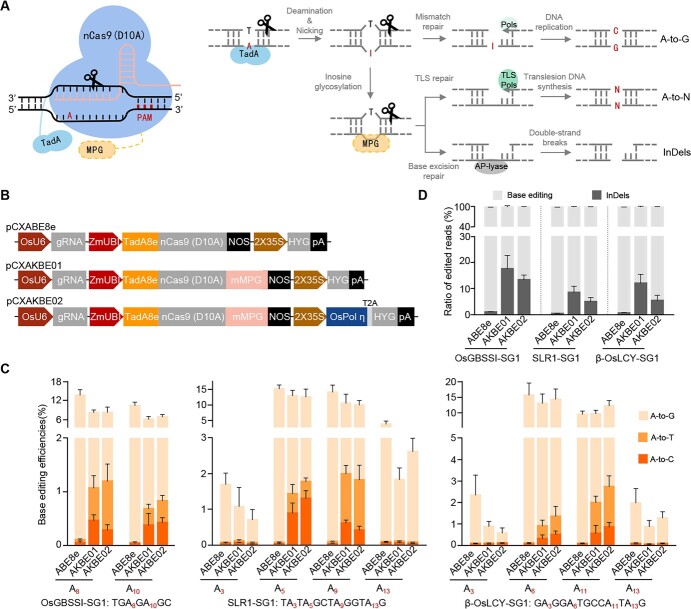
Assessment of ABE8e and AKBEs in rice protoplasts. **A** Potential pathway for adenine base editing mediated by AKBE. I, deoxyinosine; MPG, *N*-methylpurine DNA glycosylase; TLS Pols, translesion DNA synthesis polymerase; AP-lyase, abasic site lyase. **B** Diagram of ABE8e and two different AKBEs. **C** Bar plots showing A-to-G/T/C efficiencies with different adenine base editors at three endogenous targets in rice protoplasts. **D** Frequencies of editing outcomes with different adenine base editors in rice protoplasts. Bars and error bars correspond to mean and standard deviation, respectively, from three independent experiments.

A new base editor, AYBE (A-to-Y base editor), was recently reported for use in mammalian cells, enabling efficient A-to-T/C base substitution [7, 8]. In the AYBE system, the mutated human *N*-methylpurine DNA glycosylase (mMPG; Supplementary Data Fig. S1) or engineered mouse *N*-methylpurine DNA glycosylase (mAAG; Supplementary Data Fig. S1) was fused to the C-terminus of ABE. After adenine deamination to produce inosine (I), the fused MPG excises hypoxanthine (Hx) to produce an apurinic/apyrimidinic (AP) site, which leads to base replacement during DNA repair (Fig. 1A). Further engineering of the AYBE enabled the modulation of the purity of editing products in mammalian cells. By co-delivery of the translesion DNA synthesis polymerase η (TLS Polη), which preferentially incorporates A opposite AP sites [7], A-to-T editing outcomes were substantially increased [7]. In addition, Cas embedding and TadA-8e engineering significantly narrowed the editing window and increased A-to-C editing purity and efficiency [8]. In plants, generating more substitution types is beneficial for creating new germplasm resources. Recently, the AKBE (A-to-K base editor) editing systems developed in rice achieved efficient A-to-G and A-to-T editing [9, 10]. However, the heritability of the edits was not investigated, and the AKBE systems applicable to dicotyledonous plants remain to be developed. In this study, we constructed an AKBE toolkit that enabled efficient A-to-G and A-to-T editing in rice and tomato. The AKBE system could also generate A-to-C editing in rice, although the editing efficiency was not high.

## Results

### Evaluating AKBE performance in rice protoplasts

To construct AKBEs for rice, we fused the plant-codon-optimized mMPG to rABE8e [11] with a 10-amino-acid linker (SGGSGGSGGS). A bipartite nuclear localization signal peptide was fused to the C-terminus of mMPG to increase nuclear entry efficiency [12], resulting in the AKBE01 construct pCXAKBE01 (Fig. 1B). Since mMPG-induced AP sites are usually digested by AP-lyase to induce double-strand breaks (DSBs) [13], competitive binding to AP sites by overexpressed TLS polymerase may inhibit the action of endogenous AP-lyase, thus reducing DSBs (Fig. 1A). We fused rice TLS polymerase η (*OsPolη*, *Os01g0757800*) to the N-terminus of hygromycin phosphotransferase II (*HPTII*) with a viral 2A peptide to construct AKBE02 (pCXAKBE02; Fig. 1B, Supplementary Data Fig.S2).

To compare editing efficiency between ABE8e [11] and the constructed AKBE systems, we chose three endogenous targets, OsGBSSI-SG1, SLR1-SG1, and β-OsLCY-SG1 (Supplementary Data Table S2), for testing in rice protoplasts. The frequencies and ratios of different mutation types were determined by amplicon sequencing. The sequencing results demonstrated that ABE8e predominantly triggered A-to-G editing, as expected, whereas AKBE produced not only efficient A-to-G edits but also a large number of A-to-Y editing products (Fig. 1C, Supplementary Data Table S3). A-to-Y conversion mainly occurred within A5-A11 (counting the PAM position as 21–23). The amplicon sequencing results showed that AKBE01 was capable of triggering A-to-T (0.30–1.44%, on average) and A-to-C (0.29–0.89%, on average) editing at all three loci tested (Fig. 1C); it also yielded a notable percentage of InDels (averaging 1.28–1.78%; Supplementary Data Table S4). Our hypothesis posits that overexpression of rice-derived Polη may facilitate TLS repair, thereby augmenting the efficiency of A-to-Y editing (Fig. 1A). While the A-to-Y efficiencies of AKBE02 were not significantly higher than those of AKBE01 in rice protoplasts, it demonstrated considerable proficiency in inducing A-to-T (ranging from 0.41 to 1.89%, on average) and A-to-C (ranging from 0.28 to 1.30%, on average) edits (Fig. 1C). Simultaneously, the average frequencies of InDels fell within the range of 0.65–1.42% (Supplementary Data Table S4). Notably, the ratios of InDels to total edits exhibited a moderate reduction at all the tested loci compared with AKBE01 (Fig. 1D), and we chose AKBE02 for subsequent experiments in rice.

### Heritable and efficient A-to-K editing in transgenic rice

**Figure 2:**
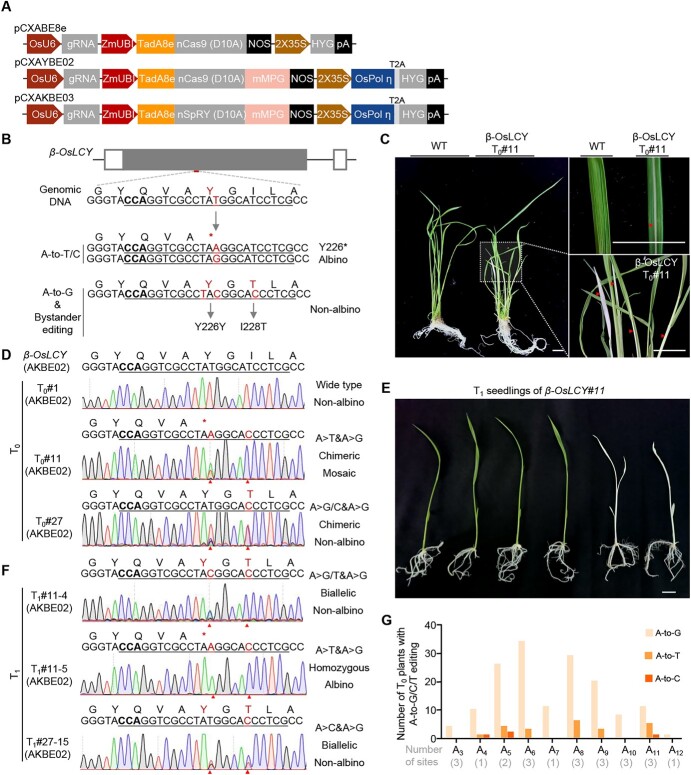
Heritable and efficient A-to-K editing induced by AKBEs in rice. **A** Schematics of the AKBE for adenine base conversion in rice. **B** Schematics to show the target at the *β-OsLCY* (*Os02g0190600*) gene and the expected editing outcomes. A-to-Y editing can change the targeted tyrosine (Y, TAT) to stop codon (TAA or TAG) and destroy the *β-OsLCY* gene, while A-to-G or bystander editing has little effect on the function of *β-OsLCY*. **C** Phenotype of *T*_0_ rice plantlets generated by AKBE02. White and pale green leaves are marked with red triangles. Scale bar, 1 cm. **D** Representative Sanger sequencing chromatograms of the target site in *β-OsLCY* with desired A-to-G, A-to-C, and A-to-T editing. Target sites are underlined, PAM sequences are highlighted in bold, and nucleotide mutations are marked with red triangles. **E** Phenotype of rice *T*_1_ progenies of *β-OsLCY*#11. White and pale green leaves are marked with red triangles. Scale bar, 1 cm. **F** Representative Sanger sequencing chromatograms of rice *T*_1_ progenies. Target sites are underlined, PAM sequences are highlighted in bold, and mutations are marked in red. **G** Summary of base-edited rice *T*_0_ plantlets across the protospacers from 11 endogenous targets; plants with edited read proportion>10% in Hi-TOM were counted as successfully edited.

We next tried to explore the feasibility of using AKBE02 in transgenic rice plants. The ABE8e and AKBE02 vectors targeting OsGBSSI-SG1, SLR1-SG1, and β-OsLCY-SG1 were each transformed into rice calli (Fig. 2A, Table 1). We first examined *T*_0_ transgenic plants using Sanger sequencing, which showed that AKBE02 produced significantly less efficient A-to-G editing compared with ABE8e. Due to the chimeric state of AKBE-generated transgenic plants, like that generated by the CGBE system [4–6], Sanger sequencing could not effectively assess the frequencies of A-to-Y (A-to-T/C). To better determine A-to-Y editing efficiencies, we further genotyped 84 plants derived from AKBE02 using Hi-TOM (chimerism rate >10% as valid edited plants; Supplementary Data Table S5) [14–15]. Sequencing results showed that 70.2% (59 out of 84) of the *T*_0_ plants contained A-to-G editing, while 17.9% (15 out of 84) and 3.6% (3 out of 84) contained A-to-T and A-to-C editing, respectively (Table 1). Consistent with the editing outcomes in protoplasts, 14 plants contained InDels (16.7%; Table 1). *β-OsLCY (Os02g0190600)* encodes lycopene β-cyclase, a key enzyme in the biosynthesis of carotenoids [16] (Fig. 2B). Mutations of *β-OsLCY* would block the carotenoid biosynthetic pathway, resulting in an albino phenotype [16]. β-OsLCY-SG1 was designed to introduce an A-to-Y substitution in *β-OsLCY*, mutating Y226 (tyrosine, TAT) to an early stop codon (TAG/TAA; Fig. 2B–D, Supplementary Data Fig.S2). However, due to the low A-to-Y editing activity, most edited *T*_0_ plants did not show an albino phenotype. Nevertheless, because of the highly chimeric state of β-OsLCY#11, mosaic albino phenotypes were observed (Fig. 2C, Supplementary Data Fig.S3).

**Table 1:** Summary of base editing efficiencies at NGG-PAM targets in *T*_0_ plantlets.

Targets	Editor	Organism	sgRNA sequence (**PAM**)	*T*_0_ plants	*T*_0_ plants containing edits			
					InDels	A-to-G	A-to-T	A-to-C
OsGBSSI-SG1	ABE8e	Rice	GGTGGTGAGAGCCGACATGG**TGG**	63	0	60 (95.2%)	0	0
SLR1-SG1			GTATAGCTAGGTAGGTTTGG**GGG**	70	0	70 (100%)	0	0
β-OsLCY-SG1			GGAGGATGCCATAGGCGACC**TGG**	76	0	76 (100%)	0	0
OsGBSSI-SG1	AKBE02		GGTGGTGAGAGCCGACATGG**TGG**	24	4 (16.7%)	14 (58.3%)	3 (12.5%)	0
SLR1-SG1			GTATAGCTAGGTAGGTTTGG**GGG**	27	4 (14.8%)	24 (88.9%)	7 (25.9%)	2 (7.4%)
β-OsLCY-SG1			GGAGGATGCCATAGGCGACC**TGG**	33	6 (18.2%)	21 (63.6%)	5 (15.2%)	1 (3%)
SlALS2-SG1	AKBE04	Tomato	GTACCGATGATTCCCAGTGG**CGG**	11	0	6 (54.5%)	0	0
SlCAO1-SG1			GTCTATGTGCACATGTGTTC**CGG**	17	2 (11.8%)	10 (58.8%)	0	0
SlCAO2-SG1			GTCTATGTGCACATGTATTC**CGG**	19	4 (21.1%)	15 (78.9%)	2 (10.5%)	0

To investigate the heritability of AKBE-generated edits, we pursued self-pollination of the *T*_0_ transgenic lines. Although the selected *T*_0_ lines were chimeric, genotyping results revealed that A-to-G, A-to-T, and A-to-C conversions could all be detected in their *T*_1_ progenies (Fig. 2E and F), even in null-segregates (Supplementary Data Table S6). The highest transmission rates of A-to-G, A-to-T, and A-to-C were 100, 54.16, and 4.16%, respectively (Supplementary Data Table S6), demonstrating the heritability of editing by our AKBE system.

To further expand the targeting scope of AKBE, we constructed AKBE03 by replacing the nSpCas9 with nSpRY (Fig. 2A), which recognizes NNN PAM [10, 17]. Eight endogenous targets within four selected genes, *OsALS1* (*Os02g0510200*), *SLR1* (*Os03g0707600*), *OsTB1* (*Os03g0706500*), and *OsBZR1* (*Os07g0580500*), were edited with AKBE03. A total of 144 *T*_0_ plants were obtained and analyzed using Hi-TOM (chimerism rate >10% as valid edited plants; Supplementary Data Table S5). At NRN PAM, apart from 60.3% (38 out of 63) *T*_0_ plants containing A-to-G edits, AKBE03 yielded an average of 9.5% (6 out of 63) A-to-T and 1.6% (1 out of 63) A-to-C editing efficiency, respectively (Table 2). However, different from nSpRY-ABE8e [2, 18], AKBE03 exhibited low editing efficiencies at NYN PAM, with 7.4% (6 out of 81) for A-to-G and 1.2% (1 out of 81) for A-to-T on average (Table 2). Together, these data show that our AKBE systems can induce efficient A-to-G and A-to-T editing within A4-A11 (Fig. 2G), and are comparable to the recently reported results [9, 10]. Importantly, the A-to-T and A-to-C edits, along with mutant phenotypes, were also observed in *T*_1_ progenies (Fig. 2E and F), persisting even in transgene-free plants through segregation (Supplementary Data Table S6).

**Table 2:** Summary of base editing efficiencies at non-NGG-PAM targets in *T*_0_ plantlets.

Target	Editor	PAM	sgRNA sequence (**PAM**)	*T*_0_ plants	*T*_0_ plants containing edits			
					InDels	A-to-G	A-to-T	A-to-C
OsALS1-SG1		NRN	GCTATGATCCCAAGTGGGGG**CGC**	20	3 (15%)	11 (55%)	1 (5%)	1 (5%)
SLR1-SG2			GGGTTGTAGTGCACGGTGTC**CGT**	14	0	14 (100%)	2 (14.3%)	0
OsTB1-SG1	AKBE03		GCTTCATGGACTTGGAGTTG**GAG**	10	1 (10%)	9 (90%)	1 (10%)	0
OsBZR1-SG1			GCGCCATGGGAGGGCGAGAG**GAT**	19	0	4 (21.1%)	2 (10.5%)	0
OsALS1-SG2	(rice)	NYN	GGATCCCAAGTGGGGGCGCA**TTC**	15	1 (6.7%)	1 (6.7%)	1 (6.7%)	0
SLR1-SG3			GTCGCCGCCACTCTCGCGGA**CTT**	17	0	0	0	0
OsBZR1-SG2			GTCGCGGCAGAGCGCCTTGA**GCA**	22	0	2 (9.1%)	0	0
OsTB1-SG2			GGTCACCCTCGCCTCGGCAA**TCA**	27	0	3 (11.1%)	0	0
SlALS2-SG2	AKBE05	NRN	GTTCCCAGTGGCGGTGCTTT**CAA**	21	0	1 (4.8%)	0	0
SlGAI1-SG1			GACCCATAGCCATCTCAAGC**TGT**	31	0	8 (25.8%)	0	0
SlALS2-SG3	(tomato)	NYN	GCCGCCACTGGGAATCATCG**GTA**	14	0	0	0	0
SlGAI1-SG2			GGCTTGAGATGGCTATGGGT**ACA**	8	0	0	0	0

### Heritable and efficient A-to-K editing in tomato

**Figure 3:**
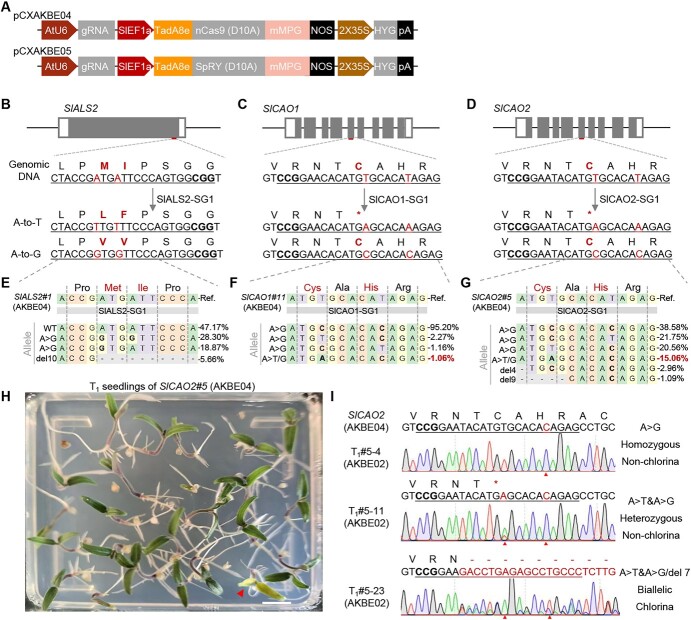
Heritable and efficient A-to-K editing induced by AKBEs in tomato. **A** Schematics of the AKBE for adenine base conversion in tomato. **B**–**D** Schematics to show the target sites at the *SlALS2* (*Solyc07g061940*) (**B**), *SlCAO1* (*Solyc06g060310*) (**C**), and *SlCAO2* (*Solyc11g012850*) (**D**) genes. At *SlCAO1* and *SlCAO2* targets, A-to-T editing can change the targeted cysteine (C, TGT) to stop codon (TGA) and destroy the *SlCAO1* and *SlCAO2* genes, but A-to-G editing would not change the cysteine (C, TGT to TGC). Mutations are marked in red. **E**–**G** Genotyping of representative edited plants at *SlALS2* (**E**), *SlCAO1* (**F**), and *SlCAO2* (**G**) targets. Frequencies of mutant alleles were determined by NGS and analyzed with CRISPResso-2.0 (CRISPResso2.pinellolab.org/). A-to-T editing (TGT to TGA) frequencies are marked in red. **H** Phenotype of *T*_1_ generations of SlCAO2#5 (AKBE04). The chlorina tomato plantlet is marked by a red triangle. Scale bar, 1 cm. **I** Representative Sanger sequencing chromatograms of tomato *T*_1_ progenies. Target sites are underlined, PAM sequences are highlighted in bold, and mutations are marked in red.

Given that the rice-optimized AKBE could produce heritable A-to-G and A-to-Y editing in rice, we next constructed a dicotyledonous AKBE system in tomato. Because both AKBE01 and AKBE02 could produce comparable A-to-Y editing in rice protoplasts (Fig. 1C), we chose AKBE01 for modification and application in tomato. Based on the excellent performance of *SlEF1α* promoter-driven ABE8e in tomato [19], we selected the *AtU6* and *SlEF1α* promoters to replace the *OsU6* and *ZmUBI* promoters of AKBE01, respectively, to construct the AKBE04 for subsequent experiments in tomato (Fig. 3A). Three loci were targeted in stable transgenic lines: SlALS2-SG1, SlCAO1-SG1, and SlCAO2-SG1 (Table 1, Fig. 3B–D). Hi-TOM sequencing of 47 *T*_0_ transgenic plants showed that AKBE04 caused highly efficient A-to-G editing (65.9% on average) at all three loci but only induced A-to-T conversion (chimerism rate >10%) at the SlCAO2-SG1 locus (2 out of 19, 10.5%; Fig. 3E–G, Table 1). Chlorophyll a oxygenase (CAO) is a Rieske-type oxygenase and is responsible for converting chlorophyll a to chlorophyll b [20, 21]. Mutations in *AtCAO* cause a yellow-green leaf phenotype [22]. Tomato has two highly conserved *CAO* genes, *SlCAO1* (*Solyc06g060310*) and *SlCAO2* (*Solyc11g012850*) (Supplementary Data Fig. S4). We designed two sgRNAs, SlCAO1-SG1 and SlCAO2-SG1, targeting the conserved C261 (cysteine, TGT) residues of these two genes. When editing occurs at the C261 position, canonical A-to-G editing (TGT to TGC) would cause a synonymous mutation, but A-to-T editing (TGT to TGA) can create nonsense mutations (Fig. 3C and D). In the *T*_0_ plants we detected A-to-T (TGT to TGA) edited alleles that disrupt the *SlCAO* genes (Fig. 3F and G). However, the edited mutants did not exhibit the chlorina phenotype due to the low rate chimerism of the A-to-T edit produced by AKBE4 in tomato (Supplementary Data Fig. S5). We self-pollinated the edited *T*_0_ tomato to produce *T*_1_ seeds. Sanger sequencing results of the *T*_1_ progenies demonstrated that the A-to-T edits were successfully inherited by the offspring (Supplementary Data Table S6), and one tomato seedling containing biallelic mutations exhibited a significant chlorina phenotype (Fig. 3H and I).

To extend the editing scope of AKBE04 in tomato, we constructed AKBE05 using nSpRY to replace nSpCas9 (Fig. 3A). We tested AKBE05 in transgenic tomato plants to edit two endogenous genes, *SlALS2* (*Solyc07g061940*) and *SlGAI* (*Solyc11g011260*), at four target sites (Supplementary Data Table S2). Seventy-four *T*_0_ plants were generated and analyzed using Hi-TOM. The results showed that AKBE05 induced efficient A-to-G editing at NRN PAM, with an average efficiency of 17.3% (Table 2, Supplementary Data TableS5). However, AKBE05 showed no detectable editing at NYN PAM in transgenic tomato (Table 2). AKBE05 was also unable to induce efficient A-to-Y editing at the targets tested (Table 2). In summary, we constructed an AKBE system in tomato to generate efficient and heritable A-to-G and A-to-T editing at NGG PAM. Nonsense mutation alleles induced by AKBE04 indicated its potential application for gene disruption (Fig. 4F and G).

## Discussion and conclusion

During the preparation of this manuscript, two studies in rice reported that the AKBE system is capable of efficient A-to-G and A-to-T editing [9, 10]. Although A-to-C editing could be produced occasionally [10], the overall efficiency was low, and the heritability of A-to-Y editing was not explored. Moreover, because the efficiency of prime editing is low in dicotyledonous plants [23, 24], the AKBE is needed in dicotyledonous plants, more so than in monocotyledonous plants [25]. Here, by optimizing AKBE in plants, we achieved A-to-G/T/C editing in rice and A-to-G/T editing in tomato. Although most of the A-to-Y edits were chimeric, we found that both the A-to-T and A-to-C edits produced by AKBE could be inherited by progeny plants through genotypic and phenotypic characterization of the *T*_1_ generation of rice and tomato plants. These results demonstrate the effectiveness of AKBE (Supplementary Data Table S3). As shown in Table 1, 15.3% (20 out of 131) edited plants contain InDels, mostly small-fragment deletions, precisely from the targeted deamination bases to Cas9 cleavage sites (Supplementary Data Table S5). This is similar to the CGBE system [5], which indicates that AKBE can also generate predictable small-fragment deletions to create genetic diversity [9, 26].

The editing results in protoplasts demonstrated that the AKBE-induced A-to-G conversions occurred within A3-A13 (Supplementary Data Table S3). In contrast, A-to-Y editing mainly occurred within A5-A11 (Supplementary Data Table S3), counting the PAM position as 21–23. As shown in Fig. 1C, the AKBE gives comparable A-to-T and A-to-C editing efficiencies in protoplasts, whereas in transgenic plants A-to-T efficiency is significantly higher than A-to-C efficiency (Tables 1 and2). This is probably due to the high number of base editors transfected and expressed in protoplasts, which induced efficient adenine deamination and hypoxanthine excision. In non-dividing protoplasts, the deoxyinosine and abasic sites may be repaired by the intrinsic DNA repair pathway but not DNA replication, which is different in the dividing callus cells and mammalian cells [27].

By targeting the third base of tyrosine (Y, TAT) or cysteine (C, TGT), A-to-T/C editing generated nonsense mutations (TAT to TAA/TAG; TGT to TGA; Supplementary Data Fig. S2). A-to-Y editing at *β-OsLCY* c.678 T can convert the Y226 residues to a stop codon (TAT to TAG), thus producing an albino phenotype. The canonical A-to-G editing at this target produces synonymous mutation, which makes this gene a useful reporter for evaluating the efficiency of AKBE-mediated A-to-T/C (not A-to-G) base substitution (Fig. 2B and C). These results also showed the potential application of this AKBE in precise gene disruption. The base-editing-induced nonsense mutations (termed CRISPR-STOP [28]) differ from commonly used NHEJ-mediated gene disruptions that rely on DSBs. As reported, the DSBs can cause unexpected genomic rearrangements and translocations [29], which are hardly detected by PCR and sequencing of target sites. Moreover, CRISPR-STOP is the preferred choice for gene therapy because of its high specificity. Therefore, further engineering of AKBE to improve its base editing purity and reduce off-target editing [7–9] will expand the scope of CRISPR-STOP.

In rice, overexpression of rice endogenous TLS Polη moderately reduced the InDel ratio (Fig. 1D), thus increasing the base editing rate and purity. Probably because TLS Polη is mainly involved in the repair of endogenous AP sites in the cell, and excess TLS Polη competitively binds to AP sites, TLS Polη overexpression inhibits the cleavage of AP sites by endogenous AP-lyase [30], thus inhibiting DNA DSBs (Fig. 1A). However, since overexpression of TLS Polη may increase random mutations in the genome [31], it would be worth exploring other ways of inhibiting AP-lyase to improve the purity of A-to-Y editing. For unknown reasons, although base editing is highly efficient in monocots such as rice, its efficiencies are still very low in dicots [32]. As reported, base editing efficiency is positively correlated with chromatin accessibility [33], and the editing efficiency of AKBE in rice can be effectively improved by fusion with the transactivation module VP64 [9]. Therefore, the editing efficiency of tomato AKBE may be enhanced by increasing chromatin accessibility, for the development of an efficient dicot-optimized AKBE system.

In summary, we developed an AKBE toolkit for rice and tomato that achieved up to 25.9 and 10.5% A-to-T editing, respectively. Notably, the desired A-to-T could be transmitted to the offspring in rice and tomato, even to transgene-free progenies. Although the A-to-C editing efficiency was not high (1.8% on average, 4 out of 228 *T*_0_ plants), it was also detected in rice *T*_1_ progenies. Subsequent Cas embedding and TadA-8e engineering [8] have the potential to improve the purity and efficiency of A-to-C editing in plants. Therefore, this study lays the foundation for further engineering the plant AYBE system. Combining plant AYBE systems with plant ABE, CBE, and CGBE, all 12 types of base conversions can be performed, which is valuable for basic plant research and genetic improvements.

## Materials and methods

### Plasmid construction

To construct the pCXAKBE01 vector, the plant codon-optimized mMPG was synthesized commercially (Genewiz, Suzhou, China) and cloned into rice rABE8e [11] by using a ClonExpress II One Step Cloning Kit (Vazyme, Nanjing, China). To construct pCXAKBE02, the OsPolη-T2A fragment was synthesized (Genewiz) and cloned into the SmaI site of pCXAKBE01. pCXAKBE03 was constructed by replacing nSpCas9 with nSpRY. nSpRY was cloned from Anc689BE4max-nSpRYCas9 [34] with primer pair SpRY-F1 + SpRY-R1. The amplified nSpRYCas9 fragment and mMPG fragment were isolated by gel purification and cloned into the SpeI/BamHI site of the pCXAKBE02 vector by using a ClonExpress II One Step Cloning Kit (Vazyme). The tomato AKBE vectors were constructed from pCXAKBE01. The *AtU6*-driven sgRNA expression cassette and *SlEF1α* promoter fragment were amplified from pSlEF1α-ABE [19] to replace the OsU6-sgRNA and *ZmUBI* promoter fragments via the HindIII/KpnI cloning site, resulting in pCXAKBE04 vector. The amplified nSpRYCas9 fragment and mMPG fragment were cloned into the SpeI/BamHI site of pCXAKBE04 to construct pCXAKBE05. The protein sequences for mMPG and OsPolη-T2A fragments are listed in Supplementary Data Table S7.

The 23-bp targeting sequences (including PAM) were selected within the target regions and their targeting specificity was analyzed using CRISPR-P 2.0 (http://crispr.hzau.edu.cn/CRISPR2/) [35]; the sgRNA expression cassettes were constructed as previously described [36]. All primers for plasmid construction are listed in Supplementary Data Table S1 and were synthesized by Sangon Biotech.

### Protoplast transfection and deep amplicon sequencing

We used the Japonica rice variety ‘Nipponbare’ to prepare the protoplasts used in this study. The rice seedlings were grown under dark conditions at 28°C for 10 days. Rice protoplast isolation and transformation were performed as described [37]. The transfected protoplasts were incubated under dark conditions at 23°C. At 48 h after transfection, the protoplasts were collected for amplicon sequencing. Genomic DNA of protoplasts was extracted with the CTAB method, and the targeted sequences were amplified with specific primers listed in Supplementary Data Table S1. The PCR products were sent for NGS sequencing (Tsingke, Beijing, China) and analyzed with CRISPResso2.0 (http://crispresso2.pinellolab.org) [38].

### Transformation of rice and tomato

For *Agrobacterium*-mediated transformation, *Agrobacterium tumefaciens* strain EHA105 was transformed with binary vectors using the freezing/heat shock method. For rice, *Agrobacterium*-mediated transformation of callus cells of ‘Nipponbare’ rice was conducted as reported [39, 40]. Hygromycin B (50 mg/l, Shanghai Yeasen Biotechnology) was used to select hygromycin-resistant calli. Plantlets were regenerated from hygromycin-resistant calli using the routine rice transformation method described previously [39, 40]. For tomato, the G17-60 cultivar was chosen for transformation. Hygromycin B (8 mg/l, Shanghai Yeasen Biotechnology) was used for selection and regeneration as described previously [23].

### Genotyping

To genotype the *T*_0_ transgenic lines, genomic DNA was extracted from leaves using the CTAB method. The amplified PCR products containing the target site were then subjected to Sanger sequencing and analyzed with DSDecodeM [41]. To further assess the mutagenesis frequency, the targeted sequences of sgRNAs were amplified for Hi-TOM sequencing and analyzed with CRISPResso2.0 (CRISPResso2.pinellolab.org/) [38]. To ensure heritability, we included only those plants with a chimerism rate >10% as valid edited plants [14, 15]. Transgene presence was checked by PCR amplification of the *HPTII* gene with primer pair HYG-F1 + HYG-R1. PCR primer sets are listed in Supplementary Data Table S1.

### Statistical analysis

The relevant statistical test, sample size, and replicate type for each figure and table are found in the figure or table and/or the corresponding figure legends.

## Supplementary Material

Web_Material_uhad250

## Data Availability

All data generated or analyzed during this study are included in this manuscript and its supplementary information files. The plasmids used in this study will be available at Addgene, and the materials are available from the corresponding author upon reasonable request.
