# Supplementary material for: Efficient and heritable A-to-K base editing in rice and tomato
Source: Hortic Res. 2023 Dec 11;11(1):uhad250. doi: 10.1093/hr/uhad250 (PMC10807703; doi:10.1093/hr/uhad250)
Supplement: Web_Material_uhad250 [file web_material_uhad250.pdf]

Supplementary figures and tables for

Efficient and heritable A-to-K base editing in rice and tomato

Xinbo Li[1,2,6], Jiyong Xie[3,4,6], Chao Dong[1,2,6], Zai Zheng[1,2], Rundong Shen[1,2], Xuesong Cao[5], Xiaoyan Chen[1], Mugui Wang[1], Jian-Kang Zhu[1,5*], Yifu Tian[1,2*]

[1]Ministry of Agriculture and Rural Affairs Key Laboratory of Gene Editing Technologies (Hainan), Institute of Crop Sciences and National Nanfan Research Institute, Chinese Academy of Agricultural Sciences, Sanya, Hainan 572024, China.

[2]Hainan Yazhou Bay Seed Lab, Sanya, Hainan 572024, China.

[3]Shanghai Center for Plant Stress Biology, Center of Excellence in Molecular Plant Sciences, Chinese Academy of Sciences, Shanghai 201602, China.

[4]University of Chinese Academy of Sciences, Beijing 100049, China.

[5]Institute of Advanced Biotechnology, and School of Life Sciences, Southern University of Science and Technology, Shenzhen 518055, China.

[6]These authors contributed equally.

Corresponding author: tianyifu@caas.cn; zhujk@sustech.edu.cn.

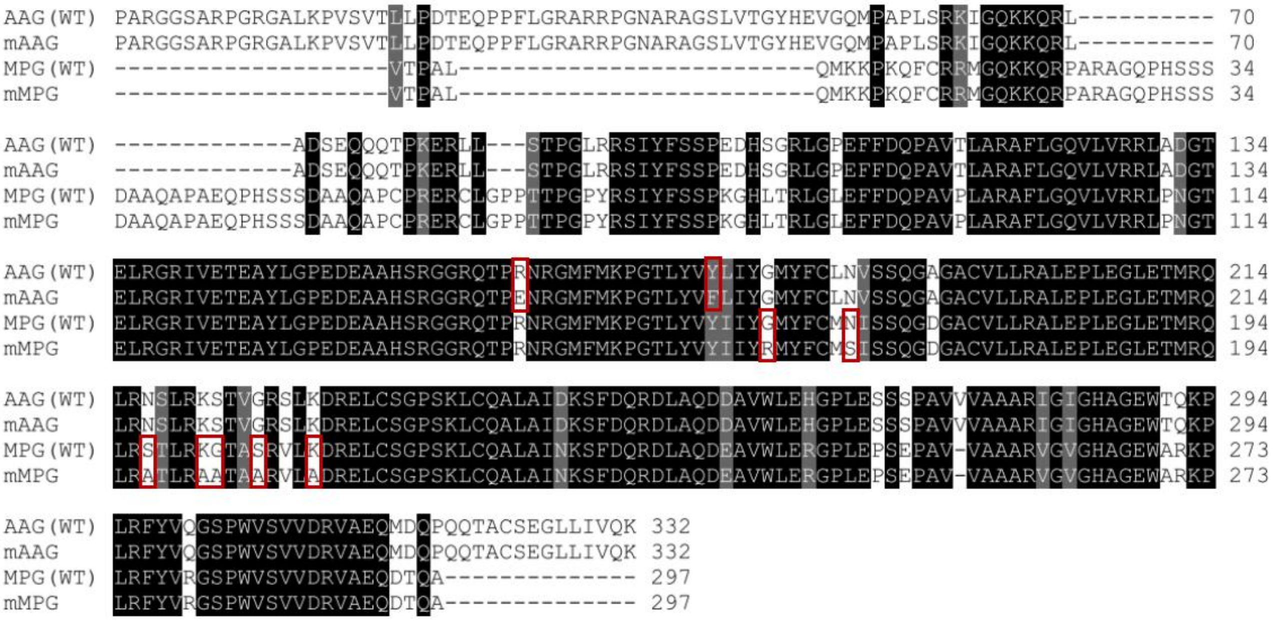

Figure S1. Sequence alignment for AAG, mAAG, MPG, and mMPG. The AAG (WT) is mouse-derived Alkyladenine DNA glycosylase, mAAG (R165E, Y179F) is an engineered version of mouse AAG. The MPG (WT) is human-derived N-methylpurine-DNA glycosylase, mMPG (G163R, N169S, S198A, K202A, G203A, S206A and K210A) is an engineered version of human MPG. The mutated amino acids were indicated with red rectangles.

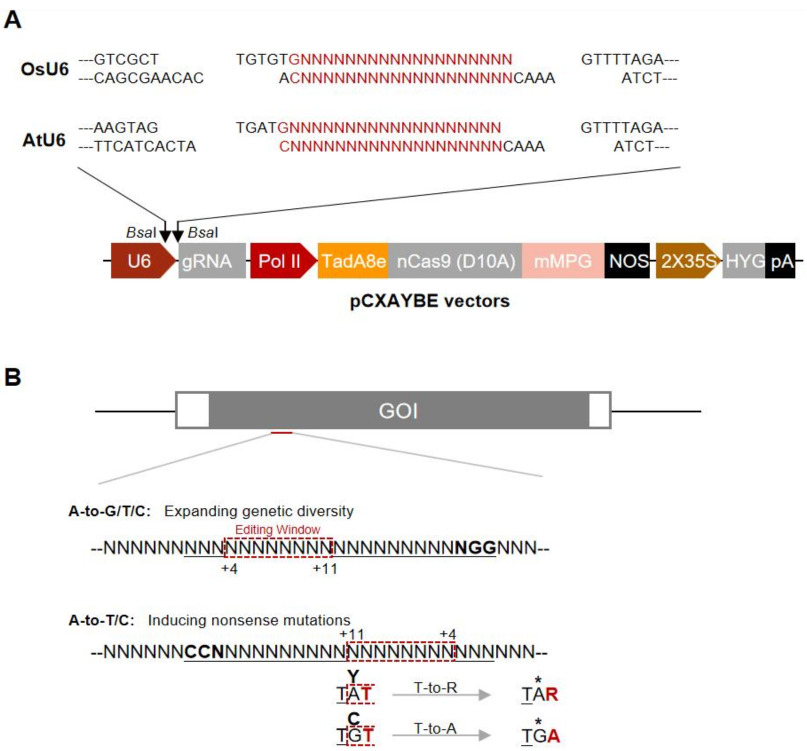

Figure S2. Schematic diagram of the construction and application of AKBE.

(A) The 20-bp targeting sequences (marked in red) were synthesized and annealed to form the oligo adaptors. AKBE vector was digested with *Bsa*I and ligated with the annealed gRNA oligos. **(B)** Potential applications of AKBE in plants. The editing windows were circled with red boxes. The sgRNAs were underlined and the PAM sequences were marked in bold. AKBE can used in expanding genetic diversity as it can produce A-to-G/T/C base conversion. Targeting the Tyrosine (Y, TAT) or Cysteine (C, TGT), A-to-T/C editing enables inducing nonsense mutations (TAT to TAA/TAG, TGT to TGA).

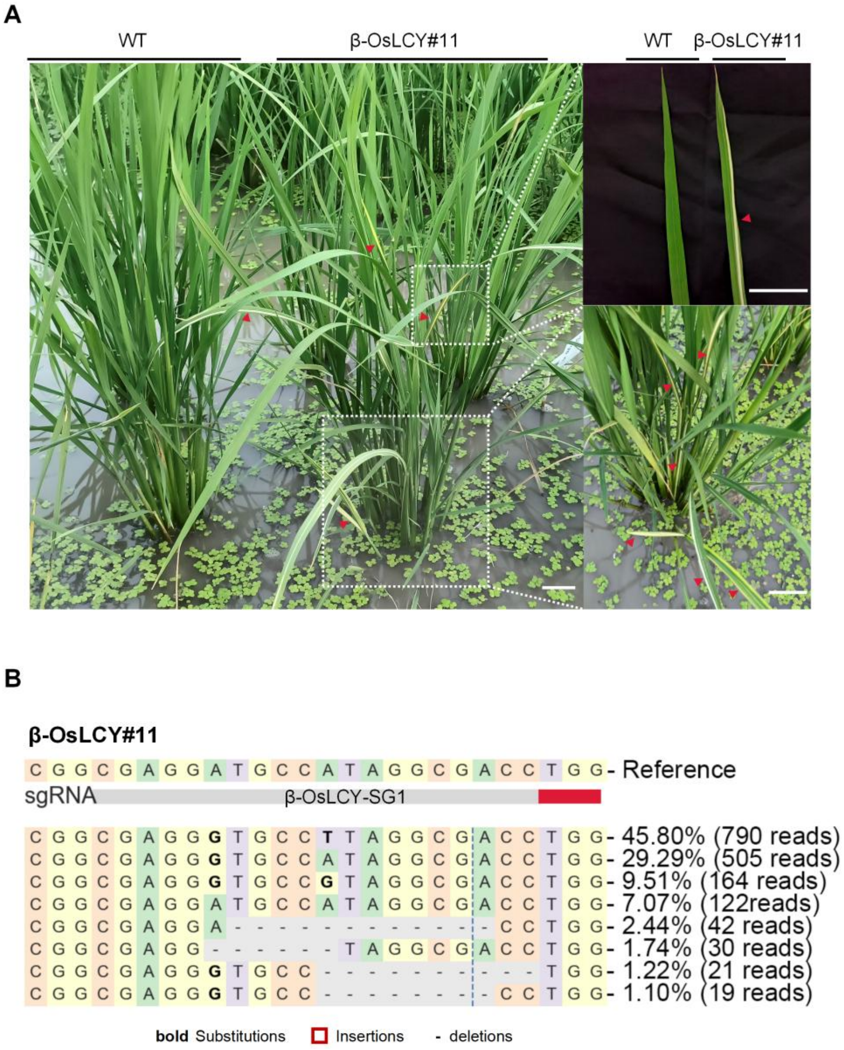

Figure S3. The phenotype of mature stage β-OsLCY#11. (A) The phenotype of β-OsLCY#11. Plants were photographed one month after transplanted into paddy soil. White and pale green leaves were marked with red triangles. Scale bar, 3 cm. **(B)** The Hi-Tom sequencing results of the β-OsLCY#11. The sgRNA was marked with a gray bar, and the PAM sequence was marked with a red bar. The base substitutions were marked in bold, and the deletion was indicated with “-”.

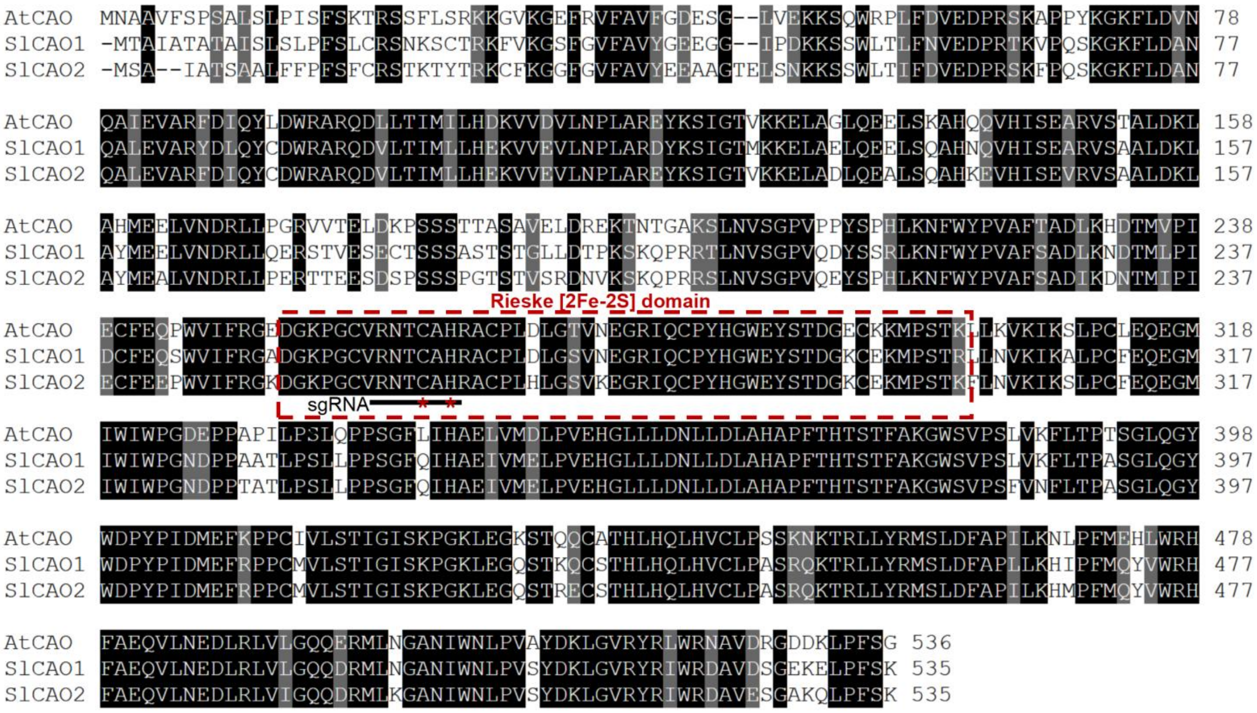

Figure S4. Sequence alignment for AtCAO, SlCAO1 and SlCAO2. The sgRNAs SlCAO1-SG1 and SlCAO2-SG1 (underlined) were both located in the Rieske [2Fe-2S] domain (circled with a red box), and the codon sequences of C261 and H263 residues were within the AKBE editing window. The C261 and H263 were marked with red stars.

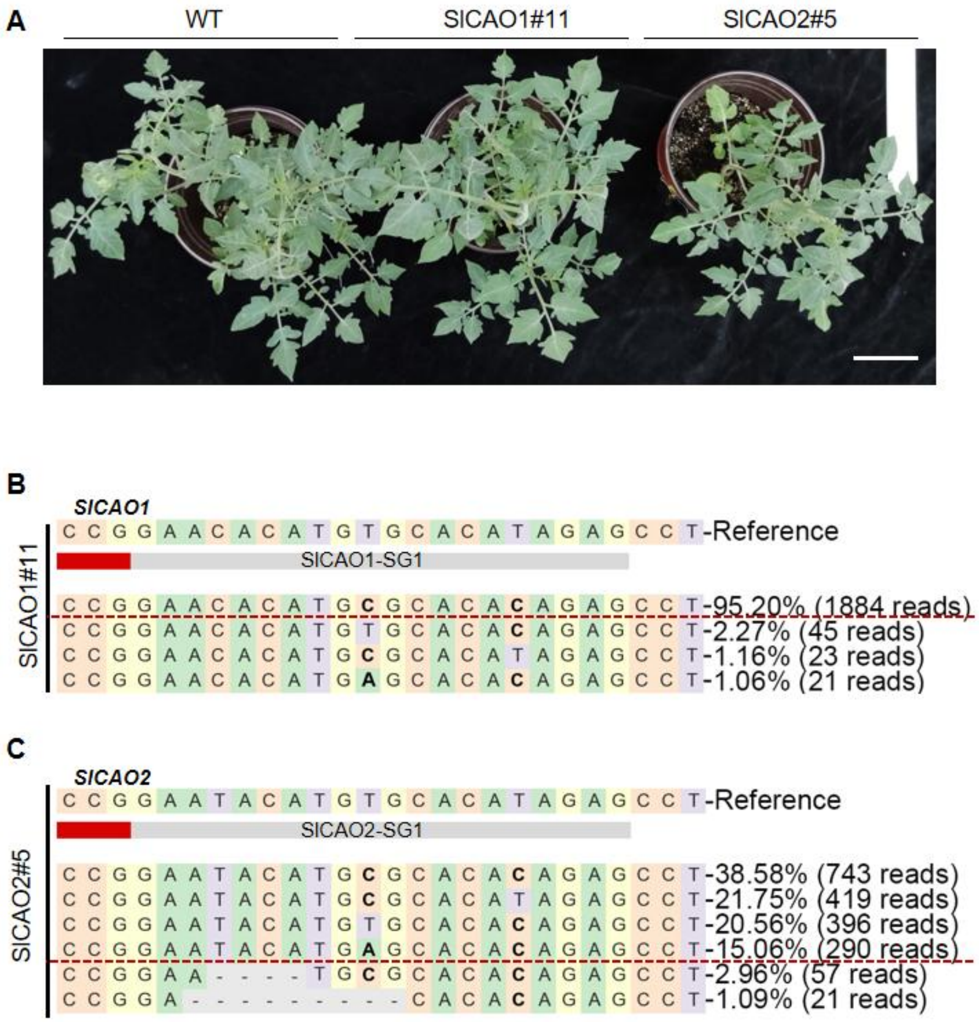

Figure S5. Representative phenotypes and genotypes of AKBE edited T_0 tomatoes. (A) Photograph of WT, SlCAO1#11 and SlCAO2#5 plants. Scale bar, 3 cm. **(B-C)** The Hi-Tom sequencing results of SlCAO1#11 **(B)** and SlCAO2#5 **(C)** mutants. The sgRNA was marked with a gray bar, and the PAM sequence was marked with a red bar. The base substitutions were marked in bold, and the deletion was indicated with “-”.

Supplementary tables

Table S1. Primers and used in this study

Primer Name	Primer Sequence	Application
OsGBSSI-HF	ggagtgagtacggtgtgcTCAATTCAGTGCAGAGATCTTC	1st PCR for Hi-TOM sequencing of OsGBSSI-SG1 target site
OsGBSSI-HR	gagttggatgctggatggGGAGCAGCGACGACGG	
OsLCY-HF	ggagtgagtacggtgtgcGCCTCGTCCAGTACGACAAG	1st PCR for Hi-TOM sequencing of OsLCY-SG1 target site
OsLCY-HR	gagttggatgctggatggAAGGGCATGGCGTAGAGGAA	
SLR1-HF	ggagtgagtacggtgtgcAACCTCGCTTCCCAACCCT	1st PCR for Hi-TOM sequencing of SLR1-SG1 target site
SLR1-HR	gagttggatgctggatggCCATCACCTTGTCCTTGCACG	
SLR1-HF2	ggagtgagtacggtgtgcTGGAGCAGCTGGAGATGGC	1st PCR for Hi-TOM sequencing of SLR1-SG2 target site
SLR1-HR2	gagttggatgctggatggCGGTGACAGTGGACGAGGT	
SLR1-HF3	ggagtgagtacggtgtgcCTTGCAGCAGGTGGGTTG	1st PCR for Hi-TOM sequencing of SLR1-SG3 target site
SLR1-HR3	gagttggatgctggatggAGTTGACGGCGATCACCTCA	
OsALS1-HF	ggagtgagtacggtgtgcAACAAAGAAGAGTGAAGTCCGT	1st PCR for Hi-TOM sequencing of OsALS1-SG1 and -SG2 target site
OsALS1-HR	gagttggatgctggatggGGTGCTTTGCCAACATACAGAT	
OsTB1-HF	ggagtgagtacggtgtgcCGCCACGATCGCCTC	1st PCR for Hi-TOM sequencing of OsTB1-SG1 target site
OsTB1-HR	gagttggatgctggatggTTATCTTGCTGTGCCGGTCCTT	
OsTB1-HF2	ggagtgagtacggtgtgcCGTCCCCGACAAGGAGTC	1st PCR for Hi-TOM sequencing of OsTB1-SG2 target site
OsTB1-HR2	gagttggatgctggatggGTGGACGATGAGTGGTTCAGG	
OsBZR1-HF	ggagtgagtacggtgtgcCCACCTACAACCTCGTCAACC	1st PCR for Hi-TOM sequencing of OsBZR1-SG1 target site
OsBZR1-HR	gagttggatgctggatggCATCTTTTTTGCTCGGCAATAATGGC	
OsBZR1-HF2	ggagtgagtacggtgtgcCGCTCGGGAACTACAACCTC	1st PCR for Hi-TOM sequencing of OsBZR1-SG2 target site
OsBZR1-HR2	gagttggatgctggatggGGCCAGATCTACACATCCAAGA	
SlALS2-HF	ggagtgagtacggtgtgcGCCATTCAAAAGATGTTAGACACTCCTGG	1st PCR for Hi-TOM sequencing of SlALS2-SG1, -SG2 target site
SlALS2-HR	gagttggatgctggatggACAATGCCTTGAACTAGTTATGTAGCTT	
SlCAO1-HF	ggagtgagtacggtgtgcTTACCGATTGATTGCTTTGAGC	1st PCR for Hi-TOM sequencing of SlCAO1-SG1 target site
SlCAO1-HR	gagttggatgctggatggCCGTGATAAGGGCATTGGATG	
SlCAO2-HF	ggagtgagtacggtgtgcGATACCAATTGAATGCTTTGAGGAAC	1st PCR for Hi-TOM sequencing of SlCAO2-SG1 target site
SlCAO2-HR	gagttggatgctggatggCCATGATAAGGGCATTGAATTCGAC	
SlGAI1-HF	ggagtgagtacggtgtgcCGAAATCGAAGATAACATACTTTGTACC	1st PCR for Hi-TOM sequencing of SlGAI1-SG1 and -SG2 target site
SlGAI1-HR	gagttggatgctggatggAGATGCTGGAATGGATGAGCT	
OsGBSSI-F1	AATGTCATATCCCCTAGCCAC	PCR for Sanger sequencing of OsGBSSI-SG1 target site
OsGBSSI-R1	GACGAACACGACGTTCATGC	
OsLCY-F1	CACCATGATGGACCGCT	PCR for Sanger sequencing of OsLCY-SG1 target site
OsLCY-R1	ACGCGTATCCCGAGGTG	
SLR1-F1	TTGCTACTACTAGTTGCTTGCCTC	PCR for Sanger sequencing of SLR1-SG1 target site
SLR1-R1	CCATGGCCATCTCCAGCTG	
mhMPG-F1	GAAGAAGAGGAAAGTCTCAGGGGGAAGTGGTGGTT	Construction of pCXAKBE01
mhMPG-R1	ATCGATCAATCAGGATCACTAGTTTACACTTTTCTTTTCTTCTTCGGCTCAAACTC	
OsPolη-F1	CTCGAGCTTTCGCAGATATGAAGAGGACAGCCGATG	Construction of pCXAKBE02
OsPolη-R1	AGAAACTTCTCGACAGATGTGGCTGTTAACTCTGGTTTCTTAGGGCCGGGATTCTC	

nSpRY-F1	GGGGCAGCAGCGGGGGATCCGACAAGAAGTACAGCATCGGCCT	Construction of pCXAKBE03 and 05
nSpRY-R1	GACTTTCCTCTTCTTCTTGGGCTCGAATT	
AtU6EF1a-F1	TTGTAAAACGACGGCCAGTG	Construction of pCXAKBE04
AtU6EF1a-R1	GCTGTCCGTTTCATGGTACCCTTGAGACACTAAGAAACTGCATTTAC	
HYG-F1	TTTATCGGCACTTTGCATCGG	Detection of transgene-free plantlets
HYG-R1	CCGTCAGGACATTGTTGGAGC	

62

Table S2. Summary of targeted loci in this study.

Targets	sgRNA sequence (**PAM**)	Gene Locus	Descriptions
OsGBSSI-SG1	GGTGGTGAGAGCCGACATGG**TGG**	Os06g0133000	Granule-bound starch synthase I
β-OsLCY-SG1	GGAGGATGCCATAGGCGACC**TGG**	Os02g0190600	Lycopene β-cyclase
SLR1-SG1	GTATAGCTAGGTAGGTTTGG**GGG**		
SLR1-SG2	GGGTTGTAGTGCACGGTGTC**CGT**	Os03g0707600	SLENDER RICE 1
SLR1-SG3	GTCGCCGCCACTCTCGCGGA**CTT**		
OsALS1-SG1	GCTATGATCCCAAGTGGGGG**CGC**	Os02g0510200	Acetolactate synthase 1
OsALS1-SG2	GGATCCCAAGTGGGGGCGCA**TTC**		
OsTB1-SG1	GCTTCATGGACTTGGAGTTG**GAG**	Os03g0706500	TEOSINTE BRANCHED 1 homolog 1
OsTB1-SG2	GGTCACCCTCGCCTCGGCAA**TCA**		
OsBZR1-SG1	GCGCCATGGGAGGGCGAGAG**GAT**	Os07g0580500	BRASSINAZOLE-RESISTANT 1 homolog 1
OsBZR1-SG2	GTCGCGGCAGAGCGCCTTGA**GCA**		
SlALS2-SG1	GTACCGATGATTCCCAGTGG**CGG**		
SlALS2-SG2	GTTCCCAGTGGCGGTGCTTT**CAA**	Solyc07g061940	Acetolactate synthase 2
SlALS2-SG3	GCCGCCACTGGGAATCATCG**GTA**		
SlCAO1-SG1	GTCTATGTGCACATGTGTTC**CGG**	Solyc06g060310	Chlorophyll a oxygenase 1
SlCAO2-SG1	GTCTATGTGCACATGTATTC**CGG**	Solyc11g012850	Chlorophyll a oxygenase 2
SlGAI1-SG1	GACCCATAGCCATCTCAAGC**TGT**	Solyc11g011260	Gibberellic acid-insensitive mutant protein 1
SlGAI1-SG2	GGCTTGAGATGGCTATGGGT**ACA**		

Table S3. The base editing types of three endogenous targets in rice protoplasts using different base editors.

	OsGBSSI-SG1		Replicate 1				Replicate 2				Replicate 3			
	**A positions**	**Editors**	**A2C**	**A2T**	**A2G**	**Total Reads**	**A2C**	**A2T**	**A2G**	**Total Reads**	**A2C**	**A2T**	**A2G**	**Total Reads**
**OsGBSSI-SG1**	**A8**	**ABE8e**	135	121	31569	206403	0	63	19420	168913	106	132	26210	172531
		**AKBE01**	757	765	10049	189799	1195	1761	14624	205012	684	960	12419	174079
		**AKBE02**	470	2236	10342	191067	677	1739	8789	171354	392	1112	17781	201748
	**A10**	**ABE8e**	127	0	21262	206403	63	0	14778	168913	0	19	19777	172531
		**AKBE01**	898	397	10536	189799	232	640	6711	205012	911	681	7510	174079
		**AKBE02**	965	601	11333	191067	744	653	6934	171354	646	1048	10966	201748

	SLR1-SG1		Replicate 1				Replicate 2				Replicate 3			
	**A positions**	**Editors**	**A2C**	**A2T**	**A2G**	**Total Reads**	**A2C**	**A2T**	**A2G**	**Total Reads**	**A2C**	**A2T**	**A2G**	**Total Reads**
**SLR1-SG1**	A_3	**ABE8e**	126	48	3271	167053	78	32	2388	181683	0	78	3038	190526
		**AKBE01**	140	98	1408	169356	81	140	2528	162955	97	43	989	203543
		**AKBE02**	38	44	1153	200618	61	97	657	174497	25	110	1973	207420
	A_5	**ABE8e**	94	30	26272	167053	49	98	28186	181683	0	92	25559	190526
		**AKBE01**	1806	648	20854	169356	1697	1388	15250	162955	1122	806	25472	203543
		**AKBE02**	2378	796	18054	200618	2021	1036	16600	174497	3228	867	28109	207420
	A_9	**ABE8e**	84	136	26272	167053	65	109	26295	181683	0	85	21711	190526
		**AKBE01**	1057	1871	20021	169356	835	2528	10775	162955	1407	3020	14521	203543
		**AKBE02**	1092	3561	16629	200618	614	1661	16601	174497	699	3098	13518	207420
	A_{13}	**ABE8e**	140	43	4940	167053	115	40	9077	181683	101	88	6976	190526
		**AKBE01**	39	112	2512	169356	135	73	2577	162955	121	63	4285	203543
		**AKBE02**	89	62	5079	200618	83	77	3766	174497	43	90	6047	207420

	β-OsLCY-SG1		Replicate 1				Replicate 2				Replicate 3			
	**A positions**	**Editors**	**A2C**	**A2T**	**A2G**	**Total Reads**	**A2C**	**A2T**	**A2G**	**Total Reads**	**A2C**	**A2T**	**A2G**	**Total Reads**
**β-OsLCY-SG1**	A_3	**ABE8e**	0	115	2318	197143	41	52	4823	161435	117	91	4830	189071
		**AKBE01**	65	84	911	182754	101	34	2068	217448	43	132	1843	207438
		**AKBE02**	106	111	1159	194904	122	32	338	213505	137	99	1233	208362
	A_6	**ABE8e**	0	137	21046	197143	116	110	29254	161435	51	40	30150	189071
		**AKBE01**	603	601	17031	182754	981	1920	22797	217448	177	1186	30988	207438
		**AKBE02**	1259	763	25053	194904	1065	1740	18911	213505	652	2778	32088	208362
	A_{11}	**ABE8e**	104	59	16901	197143	27	95	16456	161435	102	0	15321	189071
		**AKBE01**	1263	2802	10877	182754	1774	3656	17905	217448	208	2273	15583	207438
		**AKBE02**	1660	3608	13885	194904	2224	2977	19715	213505	1247	5032	21904	208362
	A_{13}	**ABE8e**	114	115	3847	197143	65	0	1864	161435	67	82	4750	189071
		**AKBE01**	0	48	2104	182754	33	124	1515	217448	25	32	1175	207438
		**AKBE02**	109	88	2948	194904	114	118	2245	213505	0	61	1996	208362

Table S4. Comparison of base edits and InDels of different base editors on three endogenous targets in rice protoplasts.

Targets	Base editors	Replicate 1			Replicate 2			Replicate 3		
		InDels	Base edits	Total reads	InDels	Base edits	Total reads	InDels	Base edits	Total reads
**OsGBSSI-SG1**	**ABE8e**	277	32004	206403	207	19611	168913	198	26679	172531
	**AKBE01**	3974	14124	189799	4145	18402	205012	2109	15660	174079
	**AKBE02**	2222	16605	191067	2418	13380	171354	3395	23866	201748
**SLR1-SG1**	**ABE8e**	40	26601	167053	60	28498	181683	122	25851	190526
	**AKBE01**	2834	25161	169356	1141	19340	162955	2959	28767	203543
	**AKBE02**	1266	22812	200618	1362	20835	174497	1102	34170	207420
**β-OsLCY-SG1**	**ABE8e**	32	21210	197143	213	29760	161435	144	30483	189071
	**AKBE01**	2765	18754	182754	4561	26474	217448	2913	33373	207438
	**AKBE02**	2120	27615	194904	1503	25440	213505	1146	36084	208362

Table S5. Genotype results of the T0 plantlets.

OsGBSSI-SG1	Mutation Sites	Alleles (>10% Hi-TOM)	Editing Type
		...GCTAGACAACCACCATGTCGGCTCTCACCACGTCCCAGCTCGCCA...	Reference
#1	WT	...GCTAGACAACCACCATGTCGGCTCTCACCACGTCCCAGCTCGCCA...	WT
#2	WT	...GCTAGACAACCACCATGTCGGCTCTCACCACGTCCCAGCTCGCCA...	A>G
	A8>G	...GCTAGACAACCACCATGTCGGCTCcCACCACGTCCCAGCTCGCCA...	
#3	WT	...GCTAGACAACCACCATGTCGGCTCTCACCACGTCCCAGCTCGCCA...	WT
#4	WT	...GCTAGACAACCACCATGTCGGCTCTCACCACGTCCCAGCTCGCCA...	WT
#5	WT	...GCTAGACAACCACCATGTCGGCTCTCACCACGTCCCAGCTCGCCA...	A>G
	A8>G	...GCTAGACAACCACCATGTCGGCTCcCACCACGTCCCAGCTCGCCA...	
#6	WT	...GCTAGACAACCACCATGTCGGCTCTCACCACGTCCCAGCTCGCCA...	WT
#7	A8>G; A10>G	...GCTAGACAACCACCATGTCGGCcCcCACCACGTCCCAGCTCGCCA...	A>G
	WT	...GCTAGACAACCACCATGTCGGCTCTCACCACGTCCCAGCTCGCCA...	
#8	A8>G	...GCTAGACAACCACCATGTCGGCTCcCACCACGTCCCAGCTCGCCA...	A>G<br>A>T
	WT	...GCTAGACAACCACCATGTCGGCTCTCACCACGTCCCAGCTCGCCA...	
	A8>T	...GCTAGACAACCACCATGTCGGCTCaCACCACGTCCCAGCTCGCCA...	
#9	WT	...GCTAGACAACCACCATGTCGGCTCTCACCACGTCCCAGCTCGCCA...	A>G<br>A>T
	A8>G	...GCTAGACAACCACCATGTCGGCTCcCACCACGTCCCAGCTCGCCA...	
	A8>T	...GCTAGACAACCACCATGTCGGCTCaCACCACGTCCCAGCTCGCCA...	
#10	WT	...GCTAGACAACCACCATGTCGGCTCTCACCACGTCCCAGCTCGCCA...	WT
#11	WT	...GCTAGACAACCACCATGTCGGCTCTCACCACGTCCCAGCTCGCCA...	A>G<br>InDels
	A8>G	...GCTAGACAACCACCATGTCGGCTCcCACCACGTCCCAGCTCGCCA...	
	A8>G; A10>G	...GCTAGACAACCACCATGTCGGCcCcCACCACGTCCCAGCTCGCCA...	
	d1	...GCTAGACAACCACCATGTCGGCTC-CACCACGTCCCAGCTCGCCA...	
#12	WT	...GCTAGACAACCACCATGTCGGCTCTCACCACGTCCCAGCTCGCCA...	WT
#13	A8>G	...GCTAGACAACCACCATGTCGGCTCcCACCACGTCCCAGCTCGCCA...	A>G
	A8>G; A10>G	...GCTAGACAACCACCATGTCGGCcCcCACCACGTCCCAGCTCGCCA...	
#14	WT	...GCTAGACAACCACCATGTCGGCTCTCACCACGTCCCAGCTCGCCA...	WT
#15	WT	...GCTAGACAACCACCATGTCGGCTCTCACCACGTCCCAGCTCGCCA...	A>G<br>InDels
	A8>G	...GCTAGACAACCACCATGTCGGCTCcCACCACGTCCCAGCTCGCCA...	
	i9	...GCTAGACAACCACCATGTCGGCTCTCtcggctctcACCACGTCCC...	
#16	WT	...GCTAGACAACCACCATGTCGGCTCTCACCACGTCCCAGCTCGCCA...	WT
#17	WT	...GCTAGACAACCACCATGTCGGCTCTCACCACGTCCCAGCTCGCCA...	WT
#18	d15	...GCTAGACAACCACCA---------------CGTCCCAGCTCGCCA...	A>G<br>InDels
	A8>G	...GCTAGACAACCACCATGTCGGCTCcCACCACGTCCCAGCTCGCCA...	
#19	A8>G	...GCTAGACAACCACCATGTCGGCTCcCACCACGTCCCAGCTCGCCA...	A>G
	A8>G; A10>G	...GCTAGACAACCACCATGTCGGCcCcCACCACGTCCCAGCTCGCCA...	
#20	WT	...GCTAGACAACCACCATGTCGGCTCTCACCACGTCCCAGCTCGCCA...	A>G<br>InDels
	d8	...GCTAGACAACCACCATG--------CACCACGTCCCAGCTCGCCA...	
	A8>G	...GCTAGACAACCACCATGTCGGCTCcCACCACGTCCCAGCTCGCCA...	
#21	A8>G	...GCTAGACAACCACCATGTCGGCTCcCACCACGTCCCAGCTCGCCA...	A>G
#22	A8>G	...GCTAGACAACCACCATGTCGGCTCcCACCACGTCCCAGCTCGCCA...	A>G
	WT	...GCTAGACAACCACCATGTCGGCTCTCACCACGTCCCAGCTCGCCA...	A>T

	A8>T; A10>G	...GCTAGACAACCACCATGTCGGCcCaCACCACGTCCCAGCTCGCCA...	
#23	WT	...GCTAGACAACCACCATGTCGGCTCTCACCACGTCCCAGCTCGCCA...	WT
#24	A8>G; A10>G	...GCTAGACAACCACCATGTCGGCcCcCACCACGTCCCAGCTCGCCA...	A>G

Table S5. Continues

SLR1-SG1	Mutation Sites	Alleles (>10% Hi-TOM)	Editing Type
		...GCAATTATTACTAGCTATAGCTAGGTAGGTTTGG**GGG**AGGCGAGA...	Reference
#1	WT	...GCAATTATTACTAGCTATAGCTAGGTAGGTTTGGGGGAGGCGAGA...	WT
#2	A5>G; A9>G	...GCAATTATTACTAGCTATgGCTgGGTAGGTTTGGGGGAGGCGAGA...	A>G
#3	WT	...GCAATTATTACTAGCTATAGCTAGGTAGGTTTGGGGGAGGCGAGA...	A>G
	A5>G	...GCAATTATTACTAGCTATgGCTAGGTAGGTTTGGGGGAGGCGAGA...	
	A5>G; A9>G	...GCAATTATTACTAGCTATgGCTgGGTAGGTTTGGGGGAGGCGAGA...	
#4	A3>G; A5>G	...GCAATTATTACTAGCTgTgGCTAGGTAGGTTTGGGGGAGGCGAGA...	A>G
	A5>G	...GCAATTATTACTAGCTATgGCTAGGTAGGTTTGGGGGAGGCGAGA...	A>T
	A3>G; A5>C; A9>T	...GCAATTATTACTAGCTgTcGCTtGGTAGGTTTGGGGGAGGCGAGA...	A>C
#5	A3>G; A5>G; A9>G	...GCAATTATTACTAGCTgTgGCTgGGTAGGTTTGGGGGAGGCGAGA...	A>G
	A5>G; A9>G	...GCAATTATTACTAGCTATgGCTgGGTAGGTTTGGGGGAGGCGAGA...	A>T
	A3>G; A5>C; A9>T	...GCAATTATTACTAGCTgTcGCTtGGTAGGTTTGGGGGAGGCGAGA...	A>C
#6	WT	...GCAATTATTACTAGCTATAGCTAGGTAGGTTTGGGGGAGGCGAGA...	A>G
	A5>T	...GCAATTATTACTAGCTATtGCTAGGTAGGTTTGGGGGAGGCGAGA...	A>T
	A5>G	...GCAATTATTACTAGCTATgGCTAGGTAGGTTTGGGGGAGGCGAGA...	
#7	A5>G; A9>G	...GCAATTATTACTAGCTATgGCTgGGTAGGTTTGGGGGAGGCGAGA...	A>G
#8	A5>G	...GCAATTATTACTAGCTATgGCTAGGTAGGTTTGGGGGAGGCGAGA...	A>G
	A5>G; A9>G	...GCAATTATTACTAGCTATgGCTgGGTAGGTTTGGGGGAGGCGAGA...	
#9	A5>G	...GCAATTATTACTAGCTATgGCTAGGTAGGTTTGGGGGAGGCGAGA...	A>G
	A5>G; A9>G	...GCAATTATTACTAGCTATgGCTgGGTAGGTTTGGGGGAGGCGAGA...	
#10	A5>G	...GCAATTATTACTAGCTATgGCTAGGTAGGTTTGGGGGAGGCGAGA...	A>G
	A5>G; A9>G	...GCAATTATTACTAGCTATgGCTgGGTAGGTTTGGGGGAGGCGAGA...	A>T
	A5>T	...GCAATTATTACTAGCTATtGCTAGGTAGGTTTGGGGGAGGCGAGA...	
#11	WT	...GCAATTATTACTAGCTATAGCTAGGTAGGTTTGGGGGAGGCGAGA...	A>G
	A5>G; A9>G	...GCAATTATTACTAGCTATgGCTgGGTAGGTTTGGGGGAGGCGAGA...	InDels
	d14	...GCAATTATTACTAGCTAT--------------GGGGGAGGCGAGA...	
#12	WT	...GCAATTATTACTAGCTATAGCTAGGTAGGTTTGGGGGAGGCGAGA...	A>G
	A5>G; A9>G	...GCAATTATTACTAGCTATgGCTgGGTAGGTTTGGGGGAGGCGAGA...	
#13	A5>G; A9>G	...GCAATTATTACTAGCTATgGCTgGGTAGGTTTGGGGGAGGCGAGA...	A>G
	A5>G	...GCAATTATTACTAGCTATgGCTAGGTAGGTTTGGGGGAGGCGAGA...	A>T
	A5>G; A9>T	...GCAATTATTACTAGCTATgGCTtGGTAGGTTTGGGGGAGGCGAGA...	
#14	d5	...GCAATTATTACTAGCTAT-----GGTAGGTTTGGGGGAGGCGAGA...	A>G
	A5>G	...GCAATTATTACTAGCTATgGCTAGGTAGGTTTGGGGGAGGCGAGA...	InDels
#15	A5>G; A9>G	...GCAATTATTACTAGCTATgGCTgGGTAGGTTTGGGGGAGGCGAGA...	A>G
	A5>T	...GCAATTATTACTAGCTATtGCTAGGTAGGTTTGGGGGAGGCGAGA...	A>T
	A5>T; A9>G	...GCAATTATTACTAGCTATtGCTgGGTAGGTTTGGGGGAGGCGAGA...	
#16	WT	...GCAATTATTACTAGCTATAGCTAGGTAGGTTTGGGGGAGGCGAGA...	WT
#17	A5>G; A9>G	...GCAATTATTACTAGCTATgGCTgGGTAGGTTTGGGGGAGGCGAGA...	A>G
	A5>T	...GCAATTATTACTAGCTATtGCTAGGTAGGTTTGGGGGAGGCGAGA...	A>T
#18	A5>G; A9>G	...GCAATTATTACTAGCTATgGCTgGGTAGGTTTGGGGGAGGCGAGA...	A>G
#19	A5>G; A9>G	...GCAATTATTACTAGCTATgGCTgGGTAGGTTTGGGGGAGGCGAGA...	A>G

	A5>G	...GCAATTATTACTAGCTATgGCTAGGTAGGTTTGGGGGAGGCGAGA...	Indels
	WT	...GCAATTATTACTAGCTATAGCTAGGTAGGTTTGGGGGAGGCGAGA...	
	d1	...GCAATTATTACTAGCTAT-GCTAGGTAGGTTTGGGGGAGGCGAGA...	
#20	WT	...GCAATTATTACTAGCTATAGCTAGGTAGGTTTGGGGGAGGCGAGA...	A>G
	A3>G; A5>G	...GCAATTATTACTAGCTgTgGCTAGGTAGGTTTGGGGGAGGCGAGA...	
	A5>G; A9>G	...GCAATTATTACTAGCTATgGCTgGGTAGGTTTGGGGGAGGCGAGA...	
#21	A5>G	...GCAATTATTACTAGCTATgGCTAGGTAGGTTTGGGGGAGGCGAGA...	A>G
	A3>G; A5>G	...GCAATTATTACTAGCTgTgGCTAGGTAGGTTTGGGGGAGGCGAGA...	
#22	WT	...GCAATTATTACTAGCTATAGCTAGGTAGGTTTGGGGGAGGCGAGA...	WT
#23	A5>G	...GCAATTATTACTAGCTATgGCTAGGTAGGTTTGGGGGAGGCGAGA...	A>G
	WT	...GCAATTATTACTAGCTATAGCTAGGTAGGTTTGGGGGAGGCGAGA...	
#24	WT	...GCAATTATTACTAGCTATAGCTAGGTAGGTTTGGGGGAGGCGAGA...	A>G
	A5>G	...GCAATTATTACTAGCTATgGCTAGGTAGGTTTGGGGGAGGCGAGA...	
	A5>G; A9>G	...GCAATTATTACTAGCTATgGCTgGGTAGGTTTGGGGGAGGCGAGA...	
#25	WT	...GCAATTATTACTAGCTATAGCTAGGTAGGTTTGGGGGAGGCGAGA...	A>G
	d11	...GCAATTATTACTAGCT-----------GGTTTGGGGGAGGCGAGA...	InDels
#26	WT	...GCAATTATTACTAGCTATAGCTAGGTAGGTTTGGGGGAGGCGAGA...	A>G
	A5>G; A9>G	...GCAATTATTACTAGCTATgGCTgGGTAGGTTTGGGGGAGGCGAGA...	
#27	A5>G	...GCAATTATTACTAGCTATgGCTAGGTAGGTTTGGGGGAGGCGAGA...	A>G
	WT	...GCAATTATTACTAGCTATAGCTAGGTAGGTTTGGGGGAGGCGAGA...	

Table S5. Continues

OsLCYB-SG1	Mutation Sites	Alleles (>10% Hi-TOM)	Editing Type
		...CGACCCGGGGTACCAGGTCGCCTATGGCATCCTCGCCGAGGTGGA...	Reference
#1	WT	...CGACCCGGGGTACCAGGTCGCCTATGGCATCCTCGCCGAGGTGGA...	WT
#2	A6>G; A11>G	...CGACCCGGGGTACCAGGTCGCCTAcGGCAcCCTCGCCGAGGTGGA...	A>G
	A6>G	...CGACCCGGGGTACCAGGTCGCCTATGGCAcCCTCGCCGAGGTGGA...	
#3	WT	...CGACCCGGGGTACCAGGTCGCCTATGGCATCCTCGCCGAGGTGGA...	WT
#4	A6>G; A11>G	...CGACCCGGGGTACCAGGTCGCCTAcGGCAcCCTCGCCGAGGTGGA...	A>G
#5	A6>G; A11>G	...CGACCCGGGGTACCAGGTCGCCTAcGGCAcCCTCGCCGAGGTGGA...	A>G<br>A>T<br>InDels
	WT	...CGACCCGGGGTACCAGGTCGCCTATGGCATCCTCGCCGAGGTGGA...	
	A6>G; A11>T	...CGACCCGGGGTACCAGGTCGCCTAaGGCAcCCTCGCCGAGGTGGA...	
	d12	...CGACCCGGGGTACCAGGTC------------CTCGCCGAGGTGGA...	
#6	WT	...CGACCCGGGGTACCAGGTCGCCTATGGCATCCTCGCCGAGGTGGA...	WT
#7	A6>G	...CGACCCGGGGTACCAGGTCGCCTATGGCAcCCTCGCCGAGGTGGA...	A>G
	A6>G; A11>T	...CGACCCGGGGTACCAGGTCGCCTAaGGCAcCCTCGCCGAGGTGGA...	A>T
#8	WT	...CGACCCGGGGTACCAGGTCGCCTATGGCATCCTCGCCGAGGTGGA...	A>G
	A6>G	...CGACCCGGGGTACCAGGTCGCCTATGGCAcCCTCGCCGAGGTGGA...	
#9	WT	...CGACCCGGGGTACCAGGTCGCCTATGGCATCCTCGCCGAGGTGGA...	A>G
	A6>G	...CGACCCGGGGTACCAGGTCGCCTATGGCAcCCTCGCCGAGGTGGA...	
	A6>G; A11>G	...CGACCCGGGGTACCAGGTCGCCTAcGGCAcCCTCGCCGAGGTGGA...	
#10	WT	...CGACCCGGGGTACCAGGTCGCCTATGGCATCCTCGCCGAGGTGGA...	A>G
	A6>G; A11>G	...CGACCCGGGGTACCAGGTCGCCTAcGGCAcCCTCGCCGAGGTGGA...	
#11	A6>G; A11>T	...CGACCCGGGGTACCAGGTCGCCTAaGGCAcCCTCGCCGAGGTGGA...	A>G
	A6>G	...CGACCCGGGGTACCAGGTCGCCTATGGCAcCCTCGCCGAGGTGGA...	A>T
#12	WT	...CGACCCGGGGTACCAGGTCGCCTATGGCATCCTCGCCGAGGTGGA...	A>G
	A6>G	...CGACCCGGGGTACCAGGTCGCCTATGGCAcCCTCGCCGAGGTGGA...	
#13	WT	...CGACCCGGGGTACCAGGTCGCCTATGGCATCCTCGCCGAGGTGGA...	WT
#14	WT	...CGACCCGGGGTACCAGGTCGCCTATGGCATCCTCGCCGAGGTGGA...	A>G
	A6>G	...CGACCCGGGGTACCAGGTCGCCTATGGCAcCCTCGCCGAGGTGGA...	
	A6>G; A11>G	...CGACCCGGGGTACCAGGTCGCCTAcGGCAcCCTCGCCGAGGTGGA...	
#15	WT	...CGACCCGGGGTACCAGGTCGCCTATGGCATCCTCGCCGAGGTGGA...	WT
#16	WT	...CGACCCGGGGTACCAGGTCGCCTATGGCATCCTCGCCGAGGTGGA...	A>G
	A6>G	...CGACCCGGGGTACCAGGTCGCCTATGGCAcCCTCGCCGAGGTGGA...	
	A6>G; A11>G	...CGACCCGGGGTACCAGGTCGCCTAcGGCAcCCTCGCCGAGGTGGA...	
#17	A6>G	...CGACCCGGGGTACCAGGTCGCCTATGGCAcCCTCGCCGAGGTGGA...	A>G
	d10	...CGACCCGGGGTACCAGGTCGCCT----------CGCCGAGGTGGA...	InDels
#18	WT	...CGACCCGGGGTACCAGGTCGCCTATGGCATCCTCGCCGAGGTGGA...	WT
#19	WT	...CGACCCGGGGTACCAGGTCGCCTATGGCATCCTCGCCGAGGTGGA...	WT
#20	WT	...CGACCCGGGGTACCAGGTCGCCTATGGCATCCTCGCCGAGGTGGA...	A>G<br>InDels
	A6>G	...CGACCCGGGGTACCAGGTCGCCTATGGCAcCCTCGCCGAGGTGGA...	
	d5	...CGACCCGGGGTACCAGGTCGCCTAT-----CCTCGCCGAGGTGGA...	
#21	A6>G	...CGACCCGGGGTACCAGGTCGCCTATGGCAcCCTCGCCGAGGTGGA...	A>G
	d5	...CGACCCGGGGTACCAGGTCGCCTAT-----CCTCGCCGAGGTGGA...	InDels

#22	WT	...CGACCCGGGGTACCAGGTCGCCTATGGCATCCTCGCCGAGGTGGA...	A>G<br>InDels
	A6>G	...CGACCCGGGGTACCAGGTCGCCTATGGCAcCCTCGCCGAGGTGGA...	
	d5	...CGACCCGGGGTACCAGGTCGCCTAT-----CCTCGCCGAGGTGGA...	
#23	WT	...CGACCCGGGGTACCAGGTCGCCTATGGCATCCTCGCCGAGGTGGA...	WT
#24	A6>G	...CGACCCGGGGTACCAGGTCGCCTATGGCAcCCTCGCCGAGGTGGA...	A>G
	A6>G; A11>G	...CGACCCGGGGTACCAGGTCGCCTAcGGCAcCCTCGCCGAGGTGGA...	
#25	WT	...CGACCCGGGGTACCAGGTCGCCTATGGCATCCTCGCCGAGGTGGA...	WT
#26	A6>G	...CGACCCGGGGTACCAGGTCGCCTATGGCAcCCTCGCCGAGGTGGA...	A>G
	A6>G; A11>G	...CGACCCGGGGTACCAGGTCGCCTAcGGCAcCCTCGCCGAGGTGGA...	
#27	WT	...CGACCCGGGGTACCAGGTCGCCTATGGCATCCTCGCCGAGGTGGA...	A>G<br>A>C
	A6>G; A11>G	...CGACCCGGGGTACCAGGTCGCCTAcGGCAcCCTCGCCGAGGTGGA...	
	A6>G; A11>C	...CGACCCGGGGTACCAGGTCGCCTAgGGCAcCCTCGCCGAGGTGGA...	
#28	WT	...CGACCCGGGGTACCAGGTCGCCTATGGCATCCTCGCCGAGGTGGA...	WT
#29	A6>G	...CGACCCGGGGTACCAGGTCGCCTATGGCAcCCTCGCCGAGGTGGA...	A>G<br>InDels
	A6>G; A11>G	...CGACCCGGGGTACCAGGTCGCCTAcGGCAcCCTCGCCGAGGTGGA...	
	d9	...CGACCCGGGGTACCAGG---------GCAcCCTCGCCGAGGTGGA...	
#30	WT	...CGACCCGGGGTACCAGGTCGCCTATGGCATCCTCGCCGAGGTGGA...	WT
#31	WT	...CGACCCGGGGTACCAGGTCGCCTATGGCATCCTCGCCGAGGTGGA...	A>G<br>A>T
	A6>G	...CGACCCGGGGTACCAGGTCGCCTATGGCAcCCTCGCCGAGGTGGA...	
	A6>G; A11>T	...CGACCCGGGGTACCAGGTCGCCTAaGGCAcCCTCGCCGAGGTGGA...	
#32	WT	...CGACCCGGGGTACCAGGTCGCCTATGGCATCCTCGCCGAGGTGGA...	A>G<br>A>T
	A6>G	...CGACCCGGGGTACCAGGTCGCCTATGGCAcCCTCGCCGAGGTGGA...	
	A6>G; A11>T	...CGACCCGGGGTACCAGGTCGCCTAaGGCAcCCTCGCCGAGGTGGA...	
#33	WT	...CGACCCGGGGTACCAGGTCGCCTATGGCATCCTCGCCGAGGTGGA...	WT

Table S5. Continues

OsALS1-SG1	Mutation Sites	Alleles (>10% Hi-TOM)	Editing Type
		...CACCAGGAGCATGTGCTGCCTATGATCCCAAGTGGGGGCGCATTC...	**Reference**
#1	WT	...CACCAGGAGCATGTGCTGCCTATGATCCCAAGTGGGGGCGCATTC...	WT
#2	WT	...CACCAGGAGCATGTGCTGCCTATGATCCCAAGTGGGGGCGCATTC...	A>G<br>A>T<br>A>C
	A4>G; A7>G	...CACCAGGAGCATGTGCTGCCTgTGgTCCCAAGTGGGGGCGCATTC...	
	A4>C; A7>G	...CACCAGGAGCATGTGCTGCCTcTGgTCCCAAGTGGGGGCGCATTC...	
	A4>T; A7>G	...CACCAGGAGCATGTGCTGCCTtTGgTCCCAAGTGGGGGCGCATTC...	
#3	WT	...CACCAGGAGCATGTGCTGCCTATGATCCCAAGTGGGGGCGCATTC...	WT
#4	WT	...CACCAGGAGCATGTGCTGCCTATGATCCCAAGTGGGGGCGCATTC...	A>G
	A4>G; A7>G	...CACCAGGAGCATGTGCTGCCTgTGgTCCCAAGTGGGGGCGCATTC...	
	A4>G; A7>G;A12>G	...CACCAGGAGCATGTGCTGCCTgTGgTCCCgAGTGGGGGCGCATTC...	
#5	WT	...CACCAGGAGCATGTGCTGCCTATGATCCCAAGTGGGGGCGCATTC...	A>G
	A4>G; A7>G	...CACCAGGAGCATGTGCTGCCTgTGgTCCCAAGTGGGGGCGCATTC...	
#6	WT	...CACCAGGAGCATGTGCTGCCTATGATCCCAAGTGGGGGCGCATTC...	WT
#7	WT	...CACCAGGAGCATGTGCTGCCTATGATCCCAAGTGGGGGCGCATTC...	WT
#8	WT	...CACCAGGAGCATGTGCTGCCTATGATCCCAAGTGGGGGCGCATTC...	A>G<br>InDels
	A7>G	...CACCAGGAGCATGTGCTGCCTATGgTCCCAAGTGGGGGCGCATTC...	
	A4>G; A7>G	...CACCAGGAGCATGTGCTGCCTgTGgTCCCAAGTGGGGGCGCATTC...	
	d4	...CACCAGGAGCATGTGCTGCCTgTG----CAAGTGGGGGCGCATTC...	
#9	WT	...CACCAGGAGCATGTGCTGCCTATGATCCCAAGTGGGGGCGCATTC...	A>G
	A4>G; A7>G	...CACCAGGAGCATGTGCTGCCTgTGgTCCCAAGTGGGGGCGCATTC...	
#10	WT	...CACCAGGAGCATGTGCTGCCTATGATCCCAAGTGGGGGCGCATTC...	WT
#11	WT	...CACCAGGAGCATGTGCTGCCTATGATCCCAAGTGGGGGCGCATTC...	A>G
	A4>G; A7>G	...CACCAGGAGCATGTGCTGCCTgTGgTCCCAAGTGGGGGCGCATTC...	
#12	WT	...CACCAGGAGCATGTGCTGCCTATGATCCCAAGTGGGGGCGCATTC...	A>G
	A4>G	...CACCAGGAGCATGTGCTGCCTgTGATCCCAAGTGGGGGCGCATTC...	
	A7>G	...CACCAGGAGCATGTGCTGCCTATGgTtCCAAGTGGGGGCGCATTC...	
	A4>G; A7>G	...CACCAGGAGCATGTGCTGCCTgTGgTCCCAAGTGGGGGCGCATTC...	
#13	WT	...CACCAGGAGCATGTGCTGCCTATGATCCCAAGTGGGGGCGCATTC...	A>G<br>InDels
	A4>G; A7>G	...CACCAGGAGCATGTGCTGCCTgTGgTCCCAAGTGGGGGCGCATTC...	
	d7	...CACCAGGAGCATGTGCTGCCT-------CAAGTGGGGGCGCATTC...	
#14	WT	...CACCAGGAGCATGTGCTGCCTATGATCCCAAGTGGGGGCGCATTC...	WT
#15	WT	...CACCAGGAGCATGTGCTGCCTATGATCCCAAGTGGGGGCGCATTC...	A>G
	A4>G	...CACCAGGAGCATGTGCTGCCTgTGATCCCAAGTGGGGGCGCATTC...	
	A4>G; A7>G	...CACCAGGAGCATGTGCTGCCTgTGgTCCCAAGTGGGGGCGCATTC...	
	A7>G	...CACCAGGAGCATGTGCTGCCTATGgTtCCAAGTGGGGGCGCATTC...	
#16	WT	...CACCAGGAGCATGTGCTGCCTATGATCCCAAGTGGGGGCGCATTC...	A>G
	A7>G	...CACCAGGAGCATGTGCTGCCTATGgTCCCAAGTGGGGGCGCATTC...	
	A4>G; A7>G	...CACCAGGAGCATGTGCTGCCTgTGgTCCCAAGTGGGGGCGCATTC...	
#17	WT	...CACCAGGAGCATGTGCTGCCTATGATCCCAAGTGGGGGCGCATTC...	WT
#18	WT	...CACCAGGAGCATGTGCTGCCTATGATCCCAAGTGGGGGCGCATTC...	WT
#19	WT	...CACCAGGAGCATGTGCTGCCTATGATCCCAAGTGGGGGCGCATTC...	A>G

	A7>G	...CACCAGGAGCATGTGCTGCCTATGgTCCCAAGTGGGGGCGCATTC...	InDels
	d10	...CACCAGGAGCATGTGCTGCCTATGgT----------GGCGCATTC...	
#20	WT	...CACCAGGAGCATGTGCTGCCTATGATCCCAAGTGGGGGCGCATTC...	WT

Table S5. Continues

SLR1-SG2	Mutation Sites	Alleles (>10% Hi-TOM)	Editing Type
		...GCACCTGGCC**ACG**GACACCGTGCACTACAACCCCTCGGACCTCTC...	**Reference**
#1	A8>G	...GCACCTGGCCACGGACACCGTGCACcACAACCCCTCGGACCTCTC...	A>G
#2	WT	...GCACCTGGCCACGGACACCGTGCACTACAACCCCTCGGACCTCTC...	A>G
	A8>G	...GCACCTGGCCACGGACACCGTGCACcACAACCCCTCGGACCTCTC...	
#3	WT	...GCACCTGGCCACGGACACCGTGCACTACAACCCCTCGGACCTCTC...	A>G<br>A>T
	A8>G	...GCACCTGGCCACGGACACCGTGCACcACAACCCCTCGGACCTCTC...	
	A8>T	...GCACCTGGCCACGGACACCGTGCACaACAACCCCTCGGACCTCTC...	
#4	A8>G	...GCACCTGGCCACGGACACCGTGCACcACAACCCCTCGGACCTCTC...	A>G
	WT	...GCACCTGGCCACGGACACCGTGCACTACAACCCCTCGGACCTCTC...	
#5	A8>G	...GCACCTGGCCACGGACACCGTGCACcACAACCCCTCGGACCTCTC...	A>G
	WT	...GCACCTGGCCACGGACACCGTGCACTACAACCCCTCGGACCTCTC...	
#6	A8>G	...GCACCTGGCCACGGACACCGTGCACcACAACCCCTCGGACCTCTC...	A>G
#7	A8>G	...GCACCTGGCCACGGACACCGTGCACcACAACCCCTCGGACCTCTC...	A>G
	A8>T	...GCACCTGGCCACGGACACCGTGCACaACAACCCCTCGGACCTCTC...	A>T
#8	A8>G	...GCACCTGGCCACGGACACCGTGCACcACAACCCCTCGGACCTCTC...	A>G
#9	A8>G	...GCACCTGGCCACGGACACCGTGCACcACAACCCCTCGGACCTCTC...	A>G
	WT	...GCACCTGGCCACGGACACCGTGCACTACAACCCCTCGGACCTCTC...	
#10	A8>G	...GCACCTGGCCACGGACACCGTGCACcACAACCCCTCGGACCTCTC...	A>G
	WT	...GCACCTGGCCACGGACACCGTGCACTACAACCCCTCGGACCTCTC...	
#11	A8>G	...GCACCTGGCCACGGACACCGTGCACcACAACCCCTCGGACCTCTC...	A>G
#12	A8>G	...GCACCTGGCCACGGACACCGTGCACcACAACCCCTCGGACCTCTC...	A>G
	WT	...GCACCTGGCCACGGACACCGTGCACTACAACCCCTCGGACCTCTC...	
#13	A8>G	...GCACCTGGCCACGGACACCGTGCACcACAACCCCTCGGACCTCTC...	A>G
	WT	...GCACCTGGCCACGGACACCGTGCACTACAACCCCTCGGACCTCTC...	
#14	A8>G	...GCACCTGGCCACGGACACCGTGCACcACAACCCCTCGGACCTCTC...	A>G

Table S5. Continues

OsTB1-SG1	Mutation Sites	Alleles (>10% Hi-TOM)	Editing Type
		...CGCCGGCCATGCACCCCTTCATGGACTTGGAGTTG**GAG**CCGCATG...	Reference
#1	WT	...CGCCGGCCATGCACCCCTTCATGGACTTGGAGTTGGAGCCGCATG...	A>G
	A6>G	...CGCCGGCCATGCACCCCTTCgTGGACTTGGAGTTGGAGCCGCATG...	
#2	A6>G	...CGCCGGCCATGCACCCCTTCgTGGACTTGGAGTTGGAGCCGCATG...	A>G
	A6>G; A10>G	...CGCCGGCCATGCACCCCTTCgTGGgCTTGGAGTTGGAGCCGCATG...	
#3	WT	...CGCCGGCCATGCACCCCTTCATGGACTTGGAGTTGGAGCCGCATG...	A>G
	A6>G	...CGCCGGCCATGCACCCCTTCgTGGACTTGGAGTTGGAGCCGCATG...	
#4	A6>G	...CGCCGGCCATGCACCCCTTCgTGGACTTGGAGTTGGAGCCGCATG...	A>G
#5	WT	...CGCCGGCCATGCACCCCTTCATGGACTTGGAGTTGGAGCCGCATG...	WT
#6	WT	...CGCCGGCCATGCACCCCTTCATGGACTTGGAGTTGGAGCCGCATG...	A>G<br>InDels
	A6>G	...CGCCGGCCATGCACCCCTTCgTGGACTTGGAGTTGGAGCCGCATG...	
	d15	...CGCCGGCCATGCACCCCTT---------------GGAGCCGCATG...	
#7	WT	...CGCCGGCCATGCACCCCTTCATGGACTTGGAGTTGGAGCCGCATG...	A>G<br>A>T
	A6>G	...CGCCGGCCATGCACCCCTTCgTGGACTTGGAGTTGGAGCCGCATG...	
	A6>T; A10>G	...CGCCGGCCATGCACCCCTTCtTGGgCTTGGAGTTGGAGCCGCATG...	
#8	WT	...CGCCGGCCATGCACCCCTTCATGGACTTGGAGTTGGAGCCGCATG...	A>G
	A6>G	...CGCCGGCCATGCACCCCTTCgTGGACTTGGAGTTGGAGCCGCATG...	
#9	WT	...CGCCGGCCATGCACCCCTTCATGGACTTGGAGTTGGAGCCGCATG...	A>G
	A6>G	...CGCCGGCCATGCACCCCTTCgTGGACTTGGAGTTGGAGCCGCATG...	
#10	WT	...CGCCGGCCATGCACCCCTTCATGGACTTGGAGTTGGAGCCGCATG...	A>G
	A6>G	...CGCCGGCCATGCACCCCTTCgTGGACTTGGAGTTGGAGCCGCATG...	

Table S5. Continues

OsBZR1-SG1	Mutation Sites	Alleles (>10% Hi-TOM)	Editing Type
		...GACAAGGGGAGGGTGACGCCATGGGAGGGCGAGAG**GAT**CCACGAG...	Reference
#1	WT	...GACAAGGGGAGGGTGACGCCATGGGAGGGCGAGAGGATCCACGAG...	WT
#2	WT	...GACAAGGGGAGGGTGACGCCATGGGAGGGCGAGAGGATCCACGAG...	WT
#3	WT	...GACAAGGGGAGGGTGACGCCATGGGAGGGCGAGAGGATCCACGAG...	WT
#4	WT	...GACAAGGGGAGGGTGACGCCATGGGAGGGCGAGAGGATCCACGAG...	WT
#5	WT	...GACAAGGGGAGGGTGACGCCATGGGAGGGCGAGAGGATCCACGAG...	A>G A>T
	A6>G	...GACAAGGGGAGGGTGACGCCgTGGGAGGGCGAGAGGATCCACGAG...	
	A6>T	...GACAAGGGGAGGGTGACGCCtTGGGAGGGCGAGAGGATCCACGAG...	
#6	WT	...GACAAGGGGAGGGTGACGCCATGGGAGGGCGAGAGGATCCACGAG...	WT
#7	WT	...GACAAGGGGAGGGTGACGCCATGGGAGGGCGAGAGGATCCACGAG...	WT
#8	WT	...GACAAGGGGAGGGTGACGCCATGGGAGGGCGAGAGGATCCACGAG...	WT
#9	WT	...GACAAGGGGAGGGTGACGCCATGGGAGGGCGAGAGGATCCACGAG...	WT
#10	WT	...GACAAGGGGAGGGTGACGCCATGGGAGGGCGAGAGGATCCACGAG...	A>G
	A6>G	...GACAAGGGGAGGGTGACGCCgTGGGAGGGCGAGAGGATCCACGAG...	
#11	WT	...GACAAGGGGAGGGTGACGCCATGGGAGGGCGAGAGGATCCACGAG...	WT
#12	WT	...GACAAGGGGAGGGTGACGCCATGGGAGGGCGAGAGGATCCACGAG...	A>G
	A6>G	...GACAAGGGGAGGGTGACGCCgTGGGAGGGCGAGAGGATCCACGAG...	
#13	WT	...GACAAGGGGAGGGTGACGCCATGGGAGGGCGAGAGGATCCACGAG...	WT
#14	WT	...GACAAGGGGAGGGTGACGCCATGGGAGGGCGAGAGGATCCACGAG...	WT
#15	WT	...GACAAGGGGAGGGTGACGCCATGGGAGGGCGAGAGGATCCACGAG...	WT
#16	WT	...GACAAGGGGAGGGTGACGCCATGGGAGGGCGAGAGGATCCACGAG...	A>G A>T
	A6>G	...GACAAGGGGAGGGTGACGCCgTGGGAGGGCGAGAGGATCCACGAG...	
	A6>T	...GACAAGGGGAGGGTGACGCCtTGGGAGGGCGAGAGGATCCACGAG...	
#17	WT	...GACAAGGGGAGGGTGACGCCATGGGAGGGCGAGAGGATCCACGAG...	WT
#18	WT	...GACAAGGGGAGGGTGACGCCATGGGAGGGCGAGAGGATCCACGAG...	WT
#19	WT	...GACAAGGGGAGGGTGACGCCATGGGAGGGCGAGAGGATCCACGAG...	WT

Table S5. Continues

OsALS1-SG2	Mutation Sites	Alleles (>10% Hi-TOM)	Editing Type
		...AGCATGTGCTGCCTATGATCCCAAGTGGGGGCGCA**TTC**AAGGACA...	Reference
#1	WT	...AGCATGTGCTGCCTATGATCCCAAGTGGGGGCGCATTCAAGGACA...	WT
#2	WT	...AGCATGTGCTGCCTATGATCCCAAGTGGGGGCGCATTCAAGGACA...	WT
#3	WT	...AGCATGTGCTGCCTATGATCCCAAGTGGGGGCGCATTCAAGGACA...	WT
#4	WT	...AGCATGTGCTGCCTATGATCCCAAGTGGGGGCGCATTCAAGGACA...	WT
#5	WT	...AGCATGTGCTGCCTATGATCCCAAGTGGGGGCGCATTCAAGGACA...	WT
#6	WT	...AGCATGTGCTGCCTATGATCCCAAGTGGGGGCGCATTCAAGGACA...	WT
#7	WT	...AGCATGTGCTGCCTATGATCCCAAGTGGGGGCGCATTCAAGGACA...	WT
#8	WT	...AGCATGTGCTGCCTATGATCCCAAGTGGGGGCGCATTCAAGGACA...	WT
#9	WT	...AGCATGTGCTGCCTATGATCCCAAGTGGGGGCGCATTCAAGGACA...	WT
#10	WT	...AGCATGTGCTGCCTATGATCCCAAGTGGGGGCGCATTCAAGGACA...	WT
#11	WT	...AGCATGTGCTGCCTATGATCCCAAGTGGGGGCGCATTCAAGGACA...	WT
#12	WT	...AGCATGTGCTGCCTATGATCCCAAGTGGGGGCGCATTCAAGGACA...	WT
#13	WT	...AGCATGTGCTGCCTATGATCCCAAGTGGGGGCGCATTCAAGGACA...	WT
#14	WT	...AGCATGTGCTGCCTATGATCCCAAGTGGGGGCGCATTCAAGGACA...	WT
#15	WT	...AGCATGTGCTGCCTATGATCCCAAGTGGGGGCGCATTCAAGGACA...	A>G<br>A>T<br>InDels
	A8>G; A9>G	...AGCATGTGCTGCCTATGATCCCggGTGGGGGCGCATTCAAGGACA...	
	A8>T	...AGCATGTGCTGCCTATGATCCCtAGTGGGGGCGCATTCAAGGACA...	
	A8>G	...AGCATGTGCTGCCTATGATCCCgAGTGGGGGCGCATTCAAGGACA...	
	d1	...AGCATGTGCTGCCTATGATCCC-AGTGGGGGCGCATTCAAGGACA...	
#16	WT	...AGCATGTGCTGCCTATGATCCCAAGTGGGGGCGCATTCAAGGACA...	WT
#17	WT	...AGCATGTGCTGCCTATGATCCCAAGTGGGGGCGCATTCAAGGACA...	WT

Table S5. Continues

OsBZR1-SG2	Mutation Sites	Alleles (>10% Hi-TOM)	Editing Type
		...ACGAGG**TGC**TCAAGGCGCTCTGCCGCGAGGCCGGCTGGGTTGTCG...	Reference
#1	WT	...ACGAGGTGCTCAAGGCGCTCTGCCGCGAGGCCGGCTGGGTTGTCG...	WT
#2	WT	...ACGAGGTGCTCAAGGCGCTCTGCCGCGAGGCCGGCTGGGTTGTCG...	WT
#3	WT	...ACGAGGTGCTCAAGGCGCTCTGCCGCGAGGCCGGCTGGGTTGTCG...	WT
#4	WT	...ACGAGGTGCTCAAGGCGCTCTGCCGCGAGGCCGGCTGGGTTGTCG...	WT
#5	WT	...ACGAGGTGCTCAAGGCGCTCTGCCGCGAGGCCGGCTGGGTTGTCG...	WT
#6	WT	...ACGAGGTGCTCAAGGCGCTCTGCCGCGAGGCCGGCTGGGTTGTCG...	WT
#7	WT	...ACGAGGTGCTCAAGGCGCTCTGCCGCGAGGCCGGCTGGGTTGTCG...	WT
#8	WT	...ACGAGGTGCTCAAGGCGCTCTGCCGCGAGGCCGGCTGGGTTGTCG...	WT
#9	WT	...ACGAGGTGCTCAAGGCGCTCTGCCGCGAGGCCGGCTGGGTTGTCG...	WT
#10	WT	...ACGAGGTGCTCAAGGCGCTCTGCCGCGAGGCCGGCTGGGTTGTCG...	A>G
	A9>G	...ACGAGGTGCTCAAGGCGCTCcGCCGCGAGGCCGGCTGGGTTGTCG...	
#11	WT	...ACGAGGTGCTCAAGGCGCTCTGCCGCGAGGCCGGCTGGGTTGTCG...	WT
#12	WT	...ACGAGGTGCTCAAGGCGCTCTGCCGCGAGGCCGGCTGGGTTGTCG...	WT
#13	WT	...ACGAGGTGCTCAAGGCGCTCTGCCGCGAGGCCGGCTGGGTTGTCG...	WT
#14	WT	...ACGAGGTGCTCAAGGCGCTCTGCCGCGAGGCCGGCTGGGTTGTCG...	WT
#15	WT	...ACGAGGTGCTCAAGGCGCTCTGCCGCGAGGCCGGCTGGGTTGTCG...	WT
#16	WT	...ACGAGGTGCTCAAGGCGCTCTGCCGCGAGGCCGGCTGGGTTGTCG...	WT
#17	WT	...ACGAGGTGCTCAAGGCGCTCTGCCGCGAGGCCGGCTGGGTTGTCG...	A>G
	A9>G	...ACGAGGTGCTCAAGGCGCTCcGCCGCGAGGCCGGCTGGGTTGTCG...	
#18	WT	...ACGAGGTGCTCAAGGCGCTCTGCCGCGAGGCCGGCTGGGTTGTCG...	WT
#19	WT	...ACGAGGTGCTCAAGGCGCTCTGCCGCGAGGCCGGCTGGGTTGTCG...	WT
#20	WT	...ACGAGGTGCTCAAGGCGCTCTGCCGCGAGGCCGGCTGGGTTGTCG...	WT
#21	WT	...ACGAGGTGCTCAAGGCGCTCTGCCGCGAGGCCGGCTGGGTTGTCG...	WT
#22	WT	...ACGAGGTGCTCAAGGCGCTCTGCCGCGAGGCCGGCTGGGTTGTCG...	WT

Table S5. Continues

OsTB1-SG2	Mutation Sites	Alleles (>10% Hi-TOM)	Editing Type
		...AAGAACCGGATGCGGTGGGTCACCCTCGCCTCGGCAA**TCA**GCGTC...	Reference
#1	WT	...AAGAACCGGATGCGGTGGGTCACCCTCGCCTCGGCAATCAGCGTC...	WT
#2	WT	...AAGAACCGGATGCGGTGGGTCACCCTCGCCTCGGCAATCAGCGTC...	WT
#3	WT	...AAGAACCGGATGCGGTGGGTCACCCTCGCCTCGGCAATCAGCGTC...	WT
#4	WT	...AAGAACCGGATGCGGTGGGTCACCCTCGCCTCGGCAATCAGCGTC...	WT
#5	WT	...AAGAACCGGATGCGGTGGGTCACCCTCGCCTCGGCAATCAGCGTC...	WT
#6	WT	...AAGAACCGGATGCGGTGGGTCACCCTCGCCTCGGCAATCAGCGTC...	WT
#7	WT	...AAGAACCGGATGCGGTGGGTCACCCTCGCCTCGGCAATCAGCGTC...	WT
#8	WT	...AAGAACCGGATGCGGTGGGTCACCCTCGCCTCGGCAATCAGCGTC...	WT
#9	WT	...AAGAACCGGATGCGGTGGGTCACCCTCGCCTCGGCAATCAGCGTC...	WT
#10	WT	...AAGAACCGGATGCGGTGGGTCACCCTCGCCTCGGCAATCAGCGTC...	WT
#11	WT	...AAGAACCGGATGCGGTGGGTCACCCTCGCCTCGGCAATCAGCGTC...	WT
#12	WT	...AAGAACCGGATGCGGTGGGTCACCCTCGCCTCGGCAATCAGCGTC...	A>G
	A5>G	...AAGAACCGGATGCGGTGGGTCgCCCTCGCCTCGGCAATCAGCGTC...	
#13	WT	...AAGAACCGGATGCGGTGGGTCACCCTCGCCTCGGCAATCAGCGTC...	WT
#14	WT	...AAGAACCGGATGCGGTGGGTCACCCTCGCCTCGGCAATCAGCGTC...	WT
#15	WT	...AAGAACCGGATGCGGTGGGTCACCCTCGCCTCGGCAATCAGCGTC...	WT
#16	WT	...AAGAACCGGATGCGGTGGGTCACCCTCGCCTCGGCAATCAGCGTC...	WT
#17	WT	...AAGAACCGGATGCGGTGGGTCACCCTCGCCTCGGCAATCAGCGTC...	A>G
	A5>G	...AAGAACCGGATGCGGTGGGTCgCCCTCGCCTCGGCAATCAGCGTC...	
#18	WT	...AAGAACCGGATGCGGTGGGTCACCCTCGCCTCGGCAATCAGCGTC...	A>G
	A5>G	...AAGAACCGGATGCGGTGGGTCgCCCTCGCCTCGGCAATCAGCGTC...	
#19	WT	...AAGAACCGGATGCGGTGGGTCACCCTCGCCTCGGCAATCAGCGTC...	WT
#20	WT	...AAGAACCGGATGCGGTGGGTCACCCTCGCCTCGGCAATCAGCGTC...	WT
#21	WT	...AAGAACCGGATGCGGTGGGTCACCCTCGCCTCGGCAATCAGCGTC...	WT
#22	WT	...AAGAACCGGATGCGGTGGGTCACCCTCGCCTCGGCAATCAGCGTC...	WT
#23	WT	...AAGAACCGGATGCGGTGGGTCACCCTCGCCTCGGCAATCAGCGTC...	WT
#24	WT	...AAGAACCGGATGCGGTGGGTCACCCTCGCCTCGGCAATCAGCGTC...	WT
#25	WT	...AAGAACCGGATGCGGTGGGTCACCCTCGCCTCGGCAATCAGCGTC...	WT
#26	WT	...AAGAACCGGATGCGGTGGGTCACCCTCGCCTCGGCAATCAGCGTC...	WT
#27	WT	...AAGAACCGGATGCGGTGGGTCACCCTCGCCTCGGCAATCAGCGTC...	WT

Table S5. Continues

SlALS2-SG1	Mutation Sites	Alleles (>10% Hi-TOM)	Editing Type
		...CATCAGGAGCATGTTCTACCGATGATTCCCAGTGG**CGG**TGCTTTC...	Reference
#1	WT	...CATCAGGAGCATGTTCTACCGATGATTCCCAGTGGCGGTGCTTTC...	A>G
	A7>G; A10>G	CATCAGGAGCATGTTCTACCGgTGgTTCCCAGTGGCGGTGCTTTC	
	A7>G	CATCAGGAGCATGTTCTACCGgTGATTCCCAGTGGCGGTGCTTTC	
#2	WT	...CATCAGGAGCATGTTCTACCGATGATTCCCAGTGGCGGTGCTTTC...	WT
#3	A7>G	...CATCAGGAGCATGTTCTACCGgTGATTCCCAGTGGCGGTGCTTTC...	A>G
	A7>G; A10>G	...CATCAGGAGCATGTTCTACCGgTGgTTCCCAGTGGCGGTGCTTTC...	
#4	WT	...CATCAGGAGCATGTTCTACCGATGATTCCCAGTGGCGGTGCTTTC...	A>G
	A7>G	...CATCAGGAGCATGTTCTACCGgTGATTCCCAGTGGCGGTGCTTTC...	
#5	WT	...CATCAGGAGCATGTTCTACCGATGATTCCCAGTGGCGGTGCTTTC...	A>G
	A7>G	...CATCAGGAGCATGTTCTACCGgTGATTCCCAGTGGCGGTGCTTTC...	
	A7>G; A10>G	...CATCAGGAGCATGTTCTACCGgTGgTTCCCAGTGGCGGTGCTTTC...	
#6	WT	...CATCAGGAGCATGTTCTACCGATGATTCCCAGTGGCGGTGCTTTC...	WT
#7	WT	...CATCAGGAGCATGTTCTACCGATGATTCCCAGTGGCGGTGCTTTC...	A>G
	A7>G	...CATCAGGAGCATGTTCTACCGgTGATTCCCAGTGGCGGTGCTTTC...	
#8	WT	...CATCAGGAGCATGTTCTACCGATGATTCCCAGTGGCGGTGCTTTC...	WT
#9	WT	...CATCAGGAGCATGTTCTACCGATGATTCCCAGTGGCGGTGCTTTC...	WT
#10	WT	...CATCAGGAGCATGTTCTACCGATGATTCCCAGTGGCGGTGCTTTC...	WT
#11	WT	...CATCAGGAGCATGTTCTACCGATGATTCCCAGTGGCGGTGCTTTC...	A>G
	A7>G	...CATCAGGAGCATGTTCTACCGgTGATTCCCAGTGGCGGTGCTTTC...	

Table S5. Continues

SlCAO1-SG1	Mutation Sites	Alleles (>10% Hi-TOM)	Editing Type
		...TGGATGTGT**CCG**GAACACATGTGCACATAGAGCCTGCCCCCTTCA...	Reference
#1	WT	...TGGATGTGTCCGGAACACATGTGCACATAGAGCCTGCCCCCTTCA...	WT
#2	WT	...TGGATGTGTCCGGAACACATGTGCACATAGAGCCTGCCCCCTTCA...	WT
#3	WT	...TGGATGTGTCCGGAACACATGTGCACATAGAGCCTGCCCCCTTCA...	A>G
	A5>G	...TGGATGTGTCCGGAACACATGTGCACAcAGAGCCTGCCCCCTTCA...	
	A5>G; A11>G	...TGGATGTGTCCGGAACACATGcGCACAcAGAGCCTGCCCCCTTCA...	
#4	WT	...TGGATGTGTCCGGAACACATGTGCACATAGAGCCTGCCCCCTTCA...	WT
#5	WT	...TGGATGTGTCCGGAACACATGTGCACATAGAGCCTGCCCCCTTCA...	A>G<br>InDels
	A5>G	...TGGATGTGTCCGGAACACATGTGCACAcAGAGCCTGCCCCCTTCA...	
	d12	...TGGATGTGTCCGGAAC------------AGAGCCTGCCCCCTTCA...	
#6	WT	...TGGATGTGTCCGGAACACATGTGCACATAGAGCCTGCCCCCTTCA...	A>G
	A5>G	...TGGATGTGTCCGGAACACATGTGCACAcAGAGCCTGCCCCCTTCA...	
#7	WT	...TGGATGTGTCCGGAACACATGTGCACATAGAGCCTGCCCCCTTCA...	WT
#8	WT	...TGGATGTGTCCGGAACACATGTGCACATAGAGCCTGCCCCCTTCA...	A>G
	A5>G	...TGGATGTGTCCGGAACACATGTGCACAcAGAGCCTGCCCCCTTCA...	
#9	WT	...TGGATGTGTCCGGAACACATGTGCACATAGAGCCTGCCCCCTTCA...	A>G
	A5>G	...TGGATGTGTCCGGAACACATGTGCACAcAGAGCCTGCCCCCTTCA...	
#10	WT	...TGGATGTGTCCGGAACACATGTGCACATAGAGCCTGCCCCCTTCA...	A>G<br>InDels
	A5>G	...TGGATGTGTCCGGAACACATGTGCACAcAGAGCCTGCCCCCTTCA...	
	d7	...TGGATGTGTCCGGAA-------GCACAcAGAGCCTGCCCCCTTCA...	
#11	A5>G; A11>G	...TGGATGTGTCCGGAACACATGcGCACAcAGAGCCTGCCCCCTTCA...	A>G
#12	WT	...TGGATGTGTCCGGAACACATGTGCACATAGAGCCTGCCCCCTTCA...	A>G
	A5>G	...TGGATGTGTCCGGAACACATGTGCACAcAGAGCCTGCCCCCTTCA...	
	A5>G; A11>G	...TGGATGTGTCCGGAACACATGcGCACAcAGAGCCTGCCCCCTTCA...	
#13	WT	...TGGATGTGTCCGGAACACATGTGCACATAGAGCCTGCCCCCTTCA...	WT
#14	A5>G	...TGGATGTGTCCGGAACACATGTGCACAcAGAGCCTGCCCCCTTCA...	A>G
	A5>G; A11>G	...TGGATGTGTCCGGAACACATGcGCACAcAGAGCCTGCCCCCTTCA...	
#15	A5>G	...TGGATGTGTCCGGAACACATGTGCACAcAGAGCCTGCCCCCTTCA...	A>G
	A5>G; A11>G	...TGGATGTGTCCGGAACACATGcGCACAcAGAGCCTGCCCCCTTCA...	
#16	WT	...TGGATGTGTCCGGAACACATGTGCACATAGAGCCTGCCCCCTTCA...	WT
#17	WT	...TGGATGTGTCCGGAACACATGTGCACATAGAGCCTGCCCCCTTCA...	WT

Table S5. Continues

SlCAO2-SG1	Mutation Sites	Alleles (>10% Hi-TOM)	Editing Type
		...TGGATGTGT**CCG**GAATACATGTGCACATAGAGCCTGCCCTCTTGA...	Reference
#1	WT	...TGGATGTGTCCGGAATACATGTGCACATAGAGCCTGCCCTCTTGA...	A>G
	A5>G	...TGGATGTGTCCGGAATACATGTGCACAcAGAGCCTGCCCTCTTGA...	
	A5>G; A11>G	...TGGATGTGTCCGGAATACATGcGCACAcAGAGCCTGCCCTCTTGA...	
#2	WT	...TGGATGTGTCCGGAATACATGTGCACATAGAGCCTGCCCTCTTGA...	WT
#3	WT	...TGGATGTGTCCGGAATACATGTGCACATAGAGCCTGCCCTCTTGA...	A>G
	A5>G	...TGGATGTGTCCGGAATACATGTGCACAcAGAGCCTGCCCTCTTGA...	
#4	WT	...TGGATGTGTCCGGAATACATGTGCACATAGAGCCTGCCCTCTTGA...	A>G
	A5>G	...TGGATGTGTCCGGAATACATGTGCACAcAGAGCCTGCCCTCTTGA...	
#5	A5>G; A11>G	...TGGATGTGTCCGGAATACATGcGCACAcAGAGCCTGCCCTCTTGA...	A>G<br>A>T
	A11>G	...TGGATGTGTCCGGAATACATGcGCACATAGAGCCTGCCCTCTTGA...	
	A5>G	...TGGATGTGTCCGGAATACATGTGCACAcAGAGCCTGCCCTCTTGA...	
	A5>G; A11>T	...TGGATGTGTCCGGAATACATGaGCACAcAGAGCCTGCCCTCTTGA...	
#6	WT	...TGGATGTGTCCGGAATACATGTGCACATAGAGCCTGCCCTCTTGA...	A>G
	A5>G	...TGGATGTGTCCGGAATACATGTGCACAcAGAGCCTGCCCTCTTGA...	
#7	WT	...TGGATGTGTCCGGAATACATGTGCACATAGAGCCTGCCCTCTTGA...	A>G<br>InDels
	A5>G	...TGGATGTGTCCGGAATACATGTGCACAcAGAGCCTGCCCTCTTGA...	
	d7	...TGGATGTGTCCGGAATACATG-------AGAGCCTGCCCTCTTGA...	
#8	WT	...TGGATGTGTCCGGAATACATGTGCACATAGAGCCTGCCCTCTTGA...	WT
#9	A5>G	...TGGATGTGTCCGGAATACATGTGCACAcAGAGCCTGCCCTCTTGA...	A>G<br>InDels
	d12	...TGGATGTGTCCGGAAT------------AGAGCCTGCCCTCTTGA...	
#10	A5>G	...TGGATGTGTCCGGAATACATGTGCACAcAGAGCCTGCCCTCTTGA...	A>G
	A5>G; A11>G	...TGGATGTGTCCGGAATACATGcGCACAcAGAGCCTGCCCTCTTGA...	
#11	WT	...TGGATGTGTCCGGAATACATGTGCACATAGAGCCTGCCCTCTTGA...	WT
#12	A5>G	...TGGATGTGTCCGGAATACATGTGCACAcAGAGCCTGCCCTCTTGA...	A>G
	A5>G; A11>G	...TGGATGTGTCCGGAATACATGcGCACAcAGAGCCTGCCCTCTTGA...	
#13	WT	...TGGATGTGTCCGGAATACATGTGCACATAGAGCCTGCCCTCTTGA...	A>G<br>InDels
	A5>G	...TGGATGTGTCCGGAATACATGTGCACAcAGAGCCTGCCCTCTTGA...	
	A5>G; A11>G	...TGGATGTGTCCGGAATACATGcGCACAcAGAGCCTGCCCTCTTGA...	
	i8	...TGGATGTGTCCGGAATACATGTGCACatgtgcacATAGAGCCTGC...	
#14	WT	...TGGATGTGTCCGGAATACATGTGCACATAGAGCCTGCCCTCTTGA...	A>G<br>A>T
	A5>G	...TGGATGTGTCCGGAATACATGTGCACAcAGAGCCTGCCCTCTTGA...	
	A5>G; A11>G	...TGGATGTGTCCGGAATACATGcGCACAcAGAGCCTGCCCTCTTGA...	
	A5>G; A11>T	...TGGATGTGTCCGGAATACATGaGCACAcAGAGCCTGCCCTCTTGA...	
#15	A5>G	...TGGATGTGTCCGGAATACATGTGCACAcAGAGCCTGCCCTCTTGA...	A>G
#16	WT	...TGGATGTGTCCGGAATACATGTGCACATAGAGCCTGCCCTCTTGA...	A>G<br>InDels
	A5>G	...TGGATGTGTCCGGAATACATGTGCACAcAGAGCCTGCCCTCTTGA...	
	d8	...TGGATGTGTCCGGA--------GCACAcAGAGCCTGCCCTCTTGA...	
#17	WT	...TGGATGTGTCCGGAATACATGTGCACATAGAGCCTGCCCTCTTGA...	WT
#18	A5>G	...TGGATGTGTCCGGAATACATGTGCACAcAGAGCCTGCCCTCTTGA...	A>G
	A5>G; A11>G	...TGGATGTGTCCGGAATACATGcGCACAcAGAGCCTGCCCTCTTGA...	

	A11>G	...TGGATGTGTCCGGAATACATGcGCACATAGAGCCTGCCCTCTTGA...	
#19	A5>G	...TGGATGTGTCCGGAATACATGcGCACAcAGAGCCTGCCCTCTTGA...	A>G

Table S5. Continues

SlALS2-SG2	Mutation Sites	Alleles (>10% Hi-TOM)	Editing Type
		...GCATGTTCTACCGATGATTCCCAGTGGCGGTGCTTT**CAA**AGATGT...	Reference
#1	WT	...GCATGTTCTACCGATGATTCCCAGTGGCGGTGCTTTCAAAGATGT...	WT
#2	WT	...GCATGTTCTACCGATGATTCCCAGTGGCGGTGCTTTCAAAGATGT...	WT
#3	WT	...GCATGTTCTACCGATGATTCCCAGTGGCGGTGCTTTCAAAGATGT...	WT
#4	WT	...GCATGTTCTACCGATGATTCCCAGTGGCGGTGCTTTCAAAGATGT...	WT
#5	WT	...GCATGTTCTACCGATGATTCCCAGTGGCGGTGCTTTCAAAGATGT...	WT
#6	WT	...GCATGTTCTACCGATGATTCCCAGTGGCGGTGCTTTCAAAGATGT...	WT
#7	WT	...GCATGTTCTACCGATGATTCCCAGTGGCGGTGCTTTCAAAGATGT...	WT
#8	WT	...GCATGTTCTACCGATGATTCCCAGTGGCGGTGCTTTCAAAGATGT...	WT
#9	WT	...GCATGTTCTACCGATGATTCCCAGTGGCGGTGCTTTCAAAGATGT...	WT
#10	WT	...GCATGTTCTACCGATGATTCCCAGTGGCGGTGCTTTCAAAGATGT...	WT
#11	WT	...GCATGTTCTACCGATGATTCCCAGTGGCGGTGCTTTCAAAGATGT...	WT
#12	WT	...GCATGTTCTACCGATGATTCCCAGTGGCGGTGCTTTCAAAGATGT...	WT
#13	WT	...GCATGTTCTACCGATGATTCCCAGTGGCGGTGCTTTCAAAGATGT...	A>G
	A7>G	...GCATGTTCTACCGATGATTCCCgGTGGCGGTGCTTTCAAAGATGT...	
#14	WT	...GCATGTTCTACCGATGATTCCCAGTGGCGGTGCTTTCAAAGATGT...	WT
#15	WT	...GCATGTTCTACCGATGATTCCCAGTGGCGGTGCTTTCAAAGATGT...	WT
#16	WT	...GCATGTTCTACCGATGATTCCCAGTGGCGGTGCTTTCAAAGATGT...	WT
#17	WT	...GCATGTTCTACCGATGATTCCCAGTGGCGGTGCTTTCAAAGATGT...	WT
#18	WT	...GCATGTTCTACCGATGATTCCCAGTGGCGGTGCTTTCAAAGATGT...	WT
#19	WT	...GCATGTTCTACCGATGATTCCCAGTGGCGGTGCTTTCAAAGATGT...	WT
#20	WT	...GCATGTTCTACCGATGATTCCCAGTGGCGGTGCTTTCAAAGATGT...	WT
#21	WT	...GCATGTTCTACCGATGATTCCCAGTGGCGGTGCTTTCAAAGATGT...	WT

Table S5. Continues

SlGAI1-SG1	Mutation Sites	Alleles (>10% Hi-TOM)	Editing Type
		...ATCTTCCATCGTTGTACCCATAGCCATCTCAAGC**TGT**TCAAGTTT...	Reference
#1	WT	...ATCTTCCATCGTTGTACCCATAGCCATCTCAAGCTGTTCAAGTTT...	A>G
	A6>G	...ATCTTCCATCGTTGTACCCgTAGCCATCTCAAGCTGTTCAAGTTT...	
	A6>G; A8>G	...ATCTTCCATCGTTGTACCCgTgGCCATCTCAAGCTGTTCAAGTTT...	
#2	WT	...ATCTTCCATCGTTGTACCCATAGCCATCTCAAGCTGTTCAAGTTT...	WT
#3	A6>G	...ATCTTCCATCGTTGTACCCgTAGCCATCTCAAGCTGTTCAAGTTT...	A>G
	A6>G; A8>G	...ATCTTCCATCGTTGTACCCgTgGCCATCTCAAGCTGTTCAAGTTT...	
#4	WT	...ATCTTCCATCGTTGTACCCATAGCCATCTCAAGCTGTTCAAGTTT...	A>G
	A6>G	...ATCTTCCATCGTTGTACCCgTAGCCATCTCAAGCTGTTCAAGTTT...	
#5	WT	...ATCTTCCATCGTTGTACCCATAGCCATCTCAAGCTGTTCAAGTTT...	WT
#6	WT	...ATCTTCCATCGTTGTACCCATAGCCATCTCAAGCTGTTCAAGTTT...	WT
#7	WT	...ATCTTCCATCGTTGTACCCATAGCCATCTCAAGCTGTTCAAGTTT...	WT
#8	WT	...ATCTTCCATCGTTGTACCCATAGCCATCTCAAGCTGTTCAAGTTT...	WT
#9	WT	...ATCTTCCATCGTTGTACCCATAGCCATCTCAAGCTGTTCAAGTTT...	WT
#10	WT	...ATCTTCCATCGTTGTACCCATAGCCATCTCAAGCTGTTCAAGTTT...	A>G
	A6>G	...ATCTTCCATCGTTGTACCCgTAGCCATCTCAAGCTGTTCAAGTTT...	
#11	WT	...ATCTTCCATCGTTGTACCCATAGCCATCTCAAGCTGTTCAAGTTT...	WT
#12	WT	...ATCTTCCATCGTTGTACCCATAGCCATCTCAAGCTGTTCAAGTTT...	WT
#13	WT	...ATCTTCCATCGTTGTACCCATAGCCATCTCAAGCTGTTCAAGTTT...	A>G
	A6>G; A8>G	...ATCTTCCATCGTTGTACCCgTgGCCATCTCAAGCTGTTCAAGTTT...	
#14	WT	...ATCTTCCATCGTTGTACCCATAGCCATCTCAAGCTGTTCAAGTTT...	WT
#15	WT	...ATCTTCCATCGTTGTACCCATAGCCATCTCAAGCTGTTCAAGTTT...	WT
#16	WT	...ATCTTCCATCGTTGTACCCATAGCCATCTCAAGCTGTTCAAGTTT...	WT
#17	WT	...ATCTTCCATCGTTGTACCCATAGCCATCTCAAGCTGTTCAAGTTT...	WT
#18	WT	...ATCTTCCATCGTTGTACCCATAGCCATCTCAAGCTGTTCAAGTTT...	WT
#19	WT	...ATCTTCCATCGTTGTACCCATAGCCATCTCAAGCTGTTCAAGTTT...	A>G
	A6>G	...ATCTTCCATCGTTGTACCCgTAGCCATCTCAAGCTGTTCAAGTTT...	
#20	WT	...ATCTTCCATCGTTGTACCCATAGCCATCTCAAGCTGTTCAAGTTT...	WT
#21	WT	...ATCTTCCATCGTTGTACCCATAGCCATCTCAAGCTGTTCAAGTTT...	WT
#22	WT	...ATCTTCCATCGTTGTACCCATAGCCATCTCAAGCTGTTCAAGTTT...	WT
#23	WT	...ATCTTCCATCGTTGTACCCATAGCCATCTCAAGCTGTTCAAGTTT...	WT
#24	WT	...ATCTTCCATCGTTGTACCCATAGCCATCTCAAGCTGTTCAAGTTT...	WT
#25	WT	...ATCTTCCATCGTTGTACCCATAGCCATCTCAAGCTGTTCAAGTTT...	A>G
	A6>G	...ATCTTCCATCGTTGTACCCgTAGCCATCTCAAGCTGTTCAAGTTT...	
#26	WT	...ATCTTCCATCGTTGTACCCATAGCCATCTCAAGCTGTTCAAGTTT...	A>G
	A6>G	...ATCTTCCATCGTTGTACCCgTAGCCATCTCAAGCTGTTCAAGTTT...	
#27	WT	...ATCTTCCATCGTTGTACCCATAGCCATCTCAAGCTGTTCAAGTTT...	WT
#28	WT	...ATCTTCCATCGTTGTACCCATAGCCATCTCAAGCTGTTCAAGTTT...	WT
#29	WT	...ATCTTCCATCGTTGTACCCATAGCCATCTCAAGCTGTTCAAGTTT...	WT
#30	WT	...ATCTTCCATCGTTGTACCCATAGCCATCTCAAGCTGTTCAAGTTT...	WT
#31	WT	...ATCTTCCATCGTTGTACCCATAGCCATCTCAAGCTGTTCAAGTTT...	WT

Table S6. Heritability analysis on T_1 progenies

Editing types	Parent			Progeny			
	Name	Generation	Hi-TOM%	Total	Base edited T_1[1]		Transmission rate
					Edited	T-DNA free[2]	
A-to-T	β-OsLCY-SG1#11	T_0	45.80%	24	13	6	54.16%
	β-OsLCY-SG1#7	T_0	13.73%	24	4	3	16.67%
	SLR1-SG1#13	T_0	18.24%	24	2	2	8.33%
	SLR1-SG1#17	T_0	10.11%	24	0	0	0.00%
	SlCAO2#5	T_0	15.06%	23	2	1	8.70%
A-to-C	β-OsLCY-SG1#27	T_0	21.37%	24	1	0	4.16%
	SLR1-SG1#4	T_0	11.75%	24	0	0	0.00%
	SLR1-SG1#5	T_0	12.33%	24	0	0	0.00%
A-to-G	β-OsLCY-SG1#11	T_0	84.60%	24	24	11	100.00%
	β-OsLCY-SG1#7	T_0	88.59%	24	24	8	100.00%
	β-OsLCY-SG1#27	T_0	66.86%	24	22	4	91.67%
	SLR1-SG1#4	T_0	91.32%	24	24	9	100.00%
	SLR1-SG1#5	T_0	90.79%	24	24	0	100.00%
	SLR1-SG1#13	T_0	96.44%	24	24	17	100.00%
	SLR1-SG1#17	T_0	83.98%	24	24	9	100.00%
	SlCAO2#5	T_0	95.95%	23	23	11	100.00%

Note.[1] The base edited T1 plants were determined by PCR and Sanger sequening. [2] The T-DNA free lines were characterized by PCR with primer pair HYG-F1+HYG-R1.

Table S7. Protein sequences of AKBE constructs.

**>AKBE.** Linker was marked in red, TadA8e in green, nSpCas9 in yellow, mhMPG in black and NLS in blue.
MKRTADGSEFESPKKKRKVSEVEFSHEYWMRHALTLAKRARDEREVPVGAVLVLNNRVIGEGWNRAIGLHDPTAHAEIMAL<br>RQGGLVMQNYRLIDATLYVTFEPCVMCAGAMIHSRIGRVVFGVRNSKRGAAGSLMNVLNYPGMNHRVEITEGILADECAAL<br>LCDFYRMPRQVFNAQKKAQSSINSGGSSGGSSGSETPGTSESATPESSGGSSGGSDKKYSIGLAIGTNSVGWAVITDEYKVPSK<br>KFKVLGNTDRHSIKKNLIGALLFDSGETAEATRLKRTARRRYTRRKNRICYLQEIFSNEMAKVDDSFFHRLEESFLVEEDKKHE<br>RHPIFGNIVDEVAYHEKYPTIYHLRKKLVDSTDKADLRLIYLALAHMIKFRGHFLIEGDLNPDNSDVDKLFIQLVQTYNQLFEE<br>NPINASGVDAKAILSARLSKSRRLENLIAQLPGEKKNGLFGNLIALSLGLTPNFKSNFDLAEDAKLQLSKDTYDDDLDNLLAQI<br>GDQYADLFLAAKNLSDAILLSDILRVNTEITKAPLSASMIKRYDEHHQDLTLLKALVRQQLPEKYKEIFFDQSKNGYAGYIDGG<br>ASQEEFYKFIKPILEKMDGTEELLVKLNREDLLRKQRTFDNGSIPHQIHLGELHAILRRQEDFYPFLKDNREKIEKILTFRIPYYV<br>GPLARGNSRFAWMTRKSEETITPWNFEEVVDKGASAQSFIERMTNFDKNLPNEKVLPKHSLLYEYFTVYNELTKVKYVTEGM<br>RKPAFLSGEQKKAIVDLLFKTNRKVTVKQLKEDYFKKIECFDSVEISGVEDRFNASLGTYHDLLKIIKDKDFLDNEENEDILED<br>IVLTLTLFEDREMIEERLKTYAHLFDDKVMKQLKRRRYTGWGRLSRKLINGIRDKQSGKTILDFLKSDGFANRNFMQLIHDDS<br>LTFKEDIQKAQVSGQGDSLHEHIANLAGSPAIKKGILQTVKVVDELVKVMGRHKPENIVIEMARENQTTQKGQKNSRERMKR<br>IEEGIKELGSQILKEHPVENTQLQNEKLYLYYLQNGRDMYVDQELDINRLSDYDVDHIVPQSFLKDDSIDNKVLTRSDKNRGK<br>SDNVPSEEVVKKMKNYWRQLLNAKLITQRKFDNLTKAERGGLSELDKAGFIKRQLVETRQITKHVAQILDSRMNTKYDEND<br>KLIREVKVITLKSKLVSDFRKDFQFYKVREINNYHHAHDAYLNAVVGTALIKKYPKLESEFVYGDYKVYDVRKMIAKSEQEIG<br>KATAKYFFYSNIMNFFKTEITLANGEIRKRPLIETNGETGEIVWDKGRDFATVRKVLSMPQVNIVKKTEVQTGGFSKESILPKR<br>NSDKLIARKKDWDPKKYGGFDSPTVAYSVLVVAKVEKGKSKKLKSVKELLGITIMERSSFEKNPIDFLEAKGYKEVKKDLIIK<br>LPKYSLFELENGRKRMLASAGELQKGNELALPSKYVNFLYLASHYEKLKGSPEDNEQKQLFVEQHKHYLDEIIEQISEFSKRVI<br>LADANLDKVLSAYNKHRDKPIREQAENIIHLFTLTNLGAPAAFKYFDTTIDRKRYTSTKEVLDATLIHQSITGLYETRIDLSQLG<br>GDSGGSKRTADGSEFEPKKKRKVSGGSGGSGGSVTPALQMKKPKQFCRRMGQKKQRPARAGQPHSSSDAAQAPAEQPHSSS<br>DAAQAPCPRERCLGPPTTPGPYRSIYFSSPKGHLTRLGLEFFDQPAVPLARAFLGQVLVRRLPNGTELRGRIVETEAYLGPEDEA<br>AHSRGGRQTPRNRGMFMKPGTLYVYIIYRMYFCMSISSQGDGACVLLRALEPLEGLETMRQLRATLRAATAARVLADRELCS<br>GPSKLCQALAINKSFDQRDLAQDEAVWLERGPLEPSEPAVVAAARVGVGHAGEWARKPLRFYVRGSPWVSVVDRVAEQDTQ<br>ASGGSKRTADGSEFEPKKKRKV*
**>OsPolη-T2A-HYG.** OsPolη was marked in red, T2A peptide in yellow, hygromycin resistance gene in black, and NLS in blue.
MKRTADGSEFESPKKKRKVPVARPEPQEPRVIAHVDMDCFYVQVEQRRNPELRGQPTAVVQYNDWKGGGLIAVSYEARKFG<br>VKRSMRGDEAKMVCPSINLVQVPVARDKADLNVYRSAGSEVVTILSTKGKCERASIDEVYLDLTDAAKEMLLESPPELLELIF<br>EEATKSNILGLPSDVSNREDSVRAWLCRADADYQDKLLSCGAIIVAQLRVKVLEETQFTCSAGIAHNKMLAKLVSGMHKPAQ<br>QTVVPSSAVQDFLVSLPIKKMKQLGGKLGSSLQDDLGVNTVGDLLSFTEDKLQEYYGVNTGTWLWKIARGISGEEVEDRLLP<br>KSHGCGKTFPGPKALKNNASVKTWLDRLCEELSERIQSDLNQNKRIAQTLTLYARACKKNKSDSIKKFPSKSCPLRYGTVKIQ<br>EDAMKLFESGLHDFLGSQNTKWSITSLSVSASKIFDIPIGTSSILRYIKGPNSTVSPANLDCSSLPEDPSLGNKLYIAPNHEEHCEP<br>SLSEKEDYGNNSNLAKQCQIKEEKKVSKKLTEVKGTCSILKFLSQSPVLSEKRKIDSLICSHPGPESSSEPNKAEEHKAAQYVD<br>RNKFNTAGSNSASSSTWMFNVEDIDPAVVEELPPEIQREIHGWIRPPKQSSSKTRGSTISSYFQPAKSGQGLMEKELRSSKGGRP<br>HNSGEGRGSLLTCGDVEENPGPKKPELTATSVEKFLIEKFDSVSDLMQLSEGEESRAFSFDVGGRGYVLRVNSCADGFYKDRY<br>VYRHFASAALPIPEVLDIGEFSESLTYCISRRAQGVTLQDLPETELPAVLQPVAEAMDAIAAADLSQTSGFGPFGPQGIGQYTTW<br>RDFICAIADPHVYHWQTVMDDTVSASVAQALDELMLWAEDCPEVRHLVHADFGSNNVLTDNGRITAVIDWSEAMFGDSQYE<br>VANIFFWRPWLACMEQQTRYFERRHPELAGSPRLRAYMLRIGLDQLYQSLVDGNFDDAAWAQGRCDAIVRSGAGTVGRTQI<br>ARRSAAVWTDGCVEVLADSGNRRPSTRPRAKK*
